# Nanoscale visualization of functional adhesion/excitability nodes at the intercalated disc

**DOI:** 10.1038/ncomms10342

**Published:** 2016-01-20

**Authors:** Alejandra Leo-Macias, Esperanza Agullo-Pascual, Jose L. Sanchez-Alonso, Sarah Keegan, Xianming Lin, Tatiana Arcos, Feng-Xia-Liang, Yuri E. Korchev, Julia Gorelik, David Fenyö, Eli Rothenberg, Mario Delmar

**Affiliations:** 1 The Leon H Charney Division of Cardiology, New York University School of Medicine (NYU-SoM), 522 First Avenue, Smilow 805, New York, New York 10016, USA; 2 Imperial College, National Heart and Lung Institute, Department of Cardiac Medicine, Imperial Center for Translational and Experimental Medicine, Hammersmith Campus, Du Cane Road, London W12 0NN, UK; 3 Center for Health Informatics and Bioinformatics, NYU-SoM, Translational Research Building, 227 East 30th Street, New York, New York 10016, USA; 4 Microscopy Core, NYU-SoM, 522 First Avenue, Skirball Institute, 2nd Floor, New York, New York 10016, USA; 5 Division of Medicine, Imperial College, Hammersmith Campus, Du Cane Road, London, London W12 0NN, UK; 6 Department of Biochemistry and Molecular Pharmacology, NYU-SoM, 522 First Avenue, MSB 3rd Floor, New York, New York 10016, USA

## Abstract

Intercellular adhesion and electrical excitability are considered separate cellular properties. Studies of myelinated fibres, however, show that voltage-gated sodium channels (VGSCs) aggregate with cell adhesion molecules at discrete subcellular locations, such as the nodes of Ranvier. Demonstration of similar macromolecular organization in cardiac muscle is missing. Here we combine nanoscale-imaging (single-molecule localization microscopy; electron microscopy; and ‘angle view' scanning patch clamp) with mathematical simulations to demonstrate distinct hubs at the cardiac intercalated disc, populated by clusters of the adhesion molecule N-cadherin and the VGSC Na_V_1.5. We show that the N-cadherin-Na_V_1.5 association is not random, that Na_V_1.5 molecules in these clusters are major contributors to cardiac sodium current, and that loss of Na_V_1.5 expression reduces intercellular adhesion strength. We speculate that adhesion/excitability nodes are key sites for crosstalk of the contractile and electrical molecular apparatus and may represent the structural substrate of cardiomyopathies in patients with mutations in molecules of the VGSC complex.

## Abstract (web summary)

In myelinated fibres conduction and adhesion proteins aggregate at discrete foci, but it is unclear if this organization is present in other excitable cells. Using nanoscale visualization and in silico techniques, the authors show that adhesion/excitability nodes exist at the intercalated discs of adult cardiac muscle.

## Graphical abstract

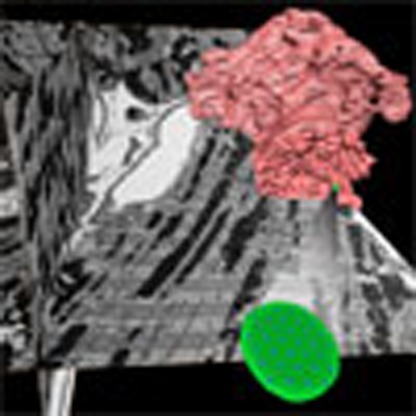

Fast sodium current is key to the generation of a rapidly conducting action potential in multiple tissues. The rapid entry of sodium into the cell depends not only from the action of one protein (the pore-forming channel subunit) but also from its integration into a macromolecular complex (the voltage-gated sodium channel (VGSC) complex). Similarly, cell adhesion is consequent to the action of multiprotein complexes that anchor the cell either to the cell matrix or to the neighbouring cell. In their classical description, cell excitability and cell adhesion are considered separate functions, carried out by independent silos of molecules. Studies in cardiac tissue preparations, however, have shown that molecules conventionally defined as belonging to the VGSC (such as the pore-forming subunit Na_V_1.5, or the scaffolding protein ankyrin-G) can co-precipitate with mechanical junction proteins^1^,^2^,^3^,^4^, and that loss of expression or mutations in specific proteins of the area composita^5^ or of the costamere lead to a reduction in sodium current amplitude^4^,^6^,^7^,^8^. Overall, the biochemical and functional data argue against the hypothesis that the VGSC is independent from cell adhesion complexes. Their functional inter-relation leads to the possibility that these complexes reside in physical proximity, and that this spatial organization is a non-random event.

In adult cardiac tissue, sodium channels organize into at least two separate pools: one at the intercalated disc (ID), and one in the cell midsection^8^,^9^,^10^. Here, we focus on the subpopulation located at the ID. This complex cellular domain, comprising several sub-domains of tightly packed clusters of molecules^5^,^11^,^12^, is highly relevant for intercellular communication and for cell excitability; in fact, sodium channels in this region are thought to carry most of the burden of propagation^13^, and a role for ID sodium channels in cell–cell propagation has also been proposed^14^,^15^,^16^. The latter hypothesis (electric field-mediated cell–cell propagation) critically depends on the actual position and organization of the sodium channels within the vast complexity of the ID structure^15^,^16^,^17^. Moreover, the proximity between adhesion and excitability complexes may play an important role in the pathogenesis of life-threatening arrhythmias associated with mutations in proteins of the ID^18^,^19^. Indeed, just as in the case of the T-tubule dyad, where nanometric separations are critical for calcium homeostasis (for example, refs 20, 21), there is an increasing recognition that in the ID molecular proximity (or separation) can greatly affect function^19^,^22^. We therefore sought to define the molecular organization of Na_V_1.5 and its position in the ID. N-cadherin, a major anatomical and functional component of the area composita in the ID plicate region, was used as a signpost to indicate the position of the main intercellular adhesion complex, to which the location of Na_V_1.5 was referred.

Studies of myelinated fibres have shown that proteins of the VGSC complex aggregate in the vicinity of adhesion molecules at discrete locations (for example, the node of Ranvier)^23^,^24^. We speculate that aggregates of adhesion and excitability molecules also exist at the ID. Yet, as opposed to the case of the node of Ranvier, an accurate molecular scale quantification of ID aggregates may be unattainable in diffraction-limited microscopy.

Therefore, to test for the presence of adhesion/excitability nodes in cardiac cells, we implement an array of nanoscale-imaging methods. The anatomical observations are complemented by functional studies to examine whether the dimensions of the Na_V_1.5 clusters are consistent with the characteristics of sodium current recorded at the nanoscale. A complete solution of the three-dimensional (3D) structure of the ID using focused ion beam scanning electron microscopy (FIB-SEM) allows us to compare the dimensionality of the observed Na_V_1.5 clusters with that of the entire ID and therefore examine whether the current magnitudes expected from the anatomical observations correspond to those previously recorded at the microscale^13^. Finally, the close physical proximity between the VGSC and the N-cadherin-rich region lead us to speculate that just as mechanical junction proteins affect the sodium current^6^,^19^,^25^, Na_V_1.5 may influence intercellular adhesion strength (IAS). These experiments unveil a novel non-canonical function for Na_V_1.5 (ref. 26), with possible implications to the understanding of the mechanisms responsible for structural heart disease associated with Na_V_1.5 mutations^27^,^28^,^29^.

## Results

### Localization of Na_V_1.5 and N-cadherin in adult heart tissue

**Figure 1:**
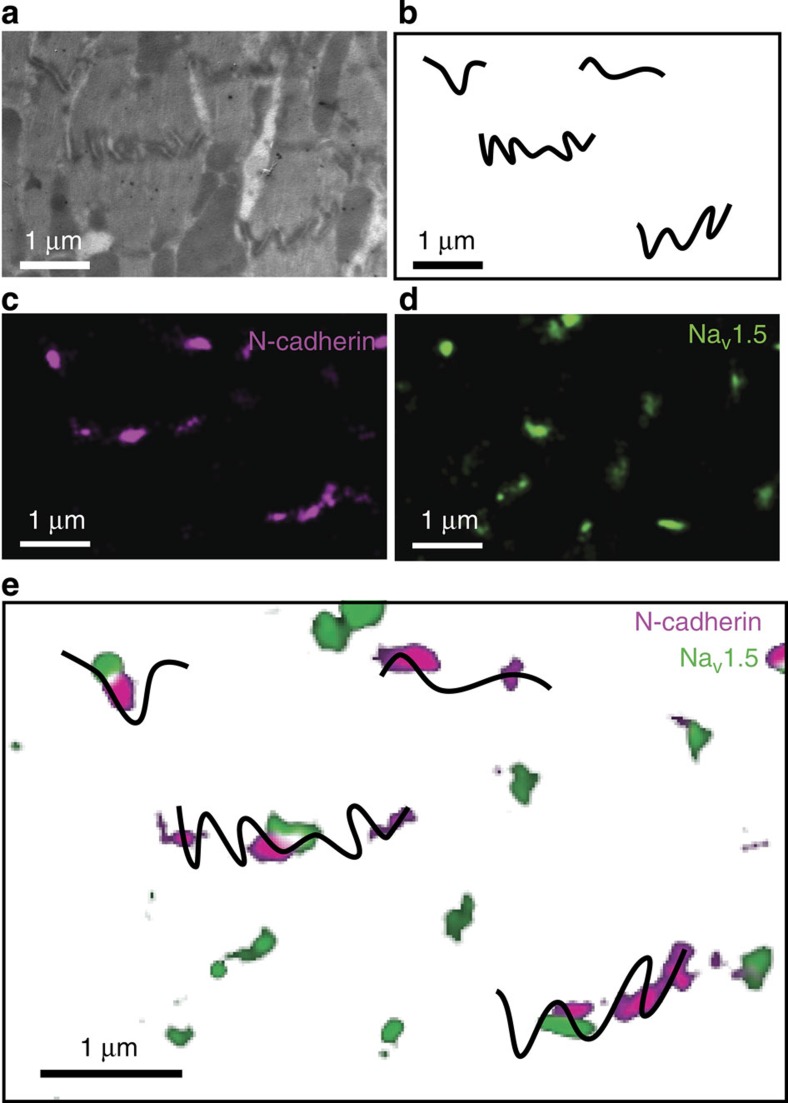
CLEM visualization of nodes populated by clusters of Na_V_1.5 and N-cadherin. EM images of areas of intercellular contact (**a**) were traced for easier visualization (**b**). Same sample was processed for detection of N-cadherin (purple; **c**) and Na_V_1.5 (green; **d**) by SMLM. The EM and SMLM images were aligned by unbiased fiducial markers (see also Supplementary Fig. 1 and Methods), allowing identification of Na_V_1.5 clusters localized to the intercellular membrane, and their proximity to N-cadherin (**e**). Scale bar, 1 μm.

**Figure 2:**
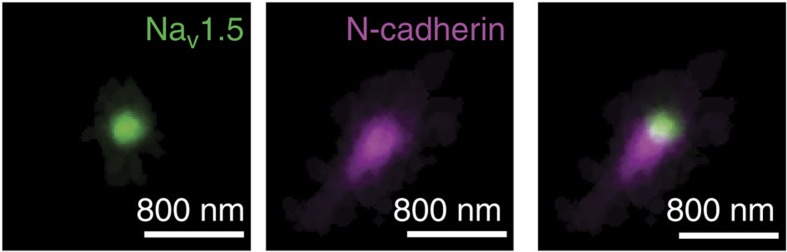
Cluster size analysis using particle averaging. Average Na_V_1.5 (green) and N-cadherin (purple) clusters derived from particle alignment and averaging analysis. (right) The spatial relation of both particles (see Methods as well as ref. 66). Only clusters where Na_V_1.5 and N-cadherin co-localized and were located on the intercellular membrane, were included in the analysis (see Supplementary Fig. 4). Scale bar, 800 nm.

**Figure 3:**
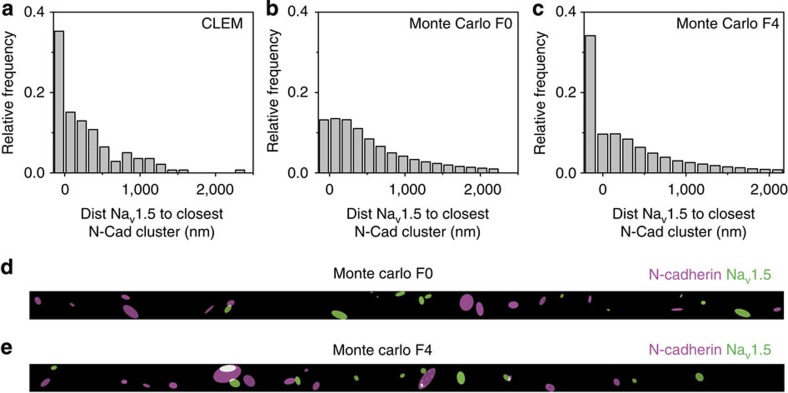
Quantification of inter-cluster distances from CLEM images. (**a**) Histogram of distances of Na_V_1.5 to nearest N-cadherin cluster; *n*=118. Data were compared with those obtained from Monte Carlo simulations modelling either random events (**b**,**d**) or a condition in which N-cadherin acted as an attractor for Na_V_1.5 (**c**,**e**). Scale bar, 1 μm.

To investigate the position of Na_V_1.5 in relation to N-Cadherin in the ID, we combined single-molecule localization microscopy (SMLM) with conventional transmission electron microscopy (TEM) in thin slices of ventricular tissue (correlative light-electron microscopy (CLEM); Supplementary Figs 1 and 2). This method allowed us to focus our attention only on those clusters of Na_V_1.5 that reside at the intercellular membrane. As expected, N-cadherin-positive signals were found mostly in segments of intercellular membranes oriented perpendicular to actin fibres (that is, plicate regions). Using dual-colour SMLM, we also localized Na_V_1.5 clusters in these preparations (Fig. 1 and Supplementary Fig. 3). A total of 118 Na_V_1.5 clusters overlaid with the electron microscopy (EM)-detected intercellular membrane. Of those, 35.2% were in contact with N-cadherin and an additional 10.8% were within 100 nm from it, with the remaining proportional fractions decreasing with distance. No clusters were found overlaying with the interplicate regions and no preference was found for a position within the width of the plicate domain. Particle averaging revealed an ellipsoid conformation of the N-cadherin clusters, with a more circular Na_V_1.5 particle that flanked, but was not surrounded by, the N-cadherin cluster (Fig. 2 and Supplementary Fig. 4). A complete frequency histogram of distances is shown in Fig. 3a (see also Supplementary Tables 1 and 2).

To assess if co-localization of Na_V_1.5 and N-cadherin was random or deterministic, we carried out Monte Carlo simulations of particle distribution in space^30^. Results are shown in Fig. 3b,c and examples of the modelled data in Fig. 3d,e. A model of purely random events failed to reproduce the experimental data (Fig. 3b,d) that, on the other hand, was mimicked by a system that established N-cadherin as an attractor for Na_V_1.5 (Fig. 3c,e; additional examples in Supplementary Fig. 5). Moreover, we speculated that Na_V_1.5 clustering would facilitate its regulation by an external modulator. As a first approach to examine this possibility, we implemented a ‘random walk' model^31^ and determined the dissociation constant (*K*_d_) of a ligand–ligate association relative to clustering of the ligand. Our results (Supplementary Fig. 6) show a significant decrease in *K*_d_ in the clustered condition. These random walk modelling results (separate and independent from the Monte Carlo simulations presented in Fig. 3) support the notion that clustering can play an important role in Na_V_1.5 regulation.

### Function and structure of Na_V_1.5 clusters at the cell end

**Figure 4:**
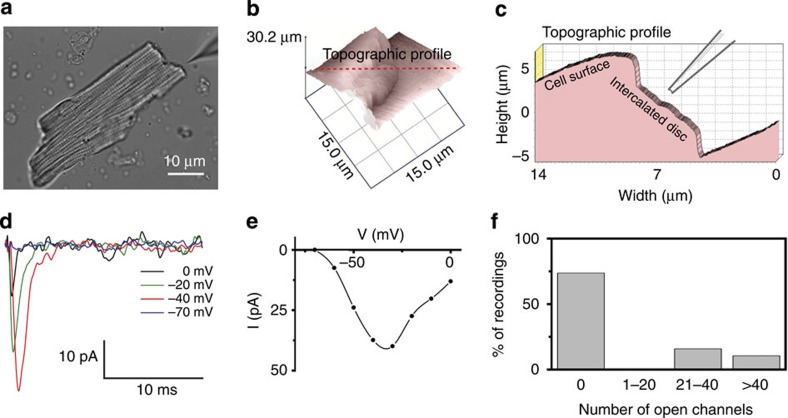
Functional analysis of Na_V_1.5 clusters in isolated mouse ventricular myocytes. (**a**) Phase contrast image of a single myocyte. A recording pipette is shown on the upper right. Scale bar, 10 μm. (**b**) Angle view SICM-generated surface topology of cell end (see also Methods; Supplementary Fig. 11). (**c**) Topographic profile of the cell in B and diagram of recording site. (**d**) Individual I_Na_ traces obtained by angle view scanning patch clamp from the cell end (voltage steps in the inset; see also Methods). (**e**) Current amplitude–voltage command relation for experiment in **d**. (**f**) Summary of independent cell-attached patch recordings. Ordinates indicate per cent of total cases where a given number of channels were detected (*n*=20).

The presence of compact Na_V_1.5 clusters at the ID observed by CLEM led us to predict that functional sodium channels in the ID would not be distributed homogenously throughout the surface but rather, as nodes of multiple channels separated by areas void of activity. We therefore implemented a new patch clamp method, based on scanning patch clamp^32^, to record channel activity in topologically defined nanodomains that are not reachable by direct orthogonal approach (‘angle view' scanning patch clamp). We resolved the structure of the cell end (Fig. 4a,b), reached its surface at a chosen site (Fig. 4c) and recorded *I*_Na_ from that specific location. A total of 20 recordings were obtained from the cell region originally forming part of the ID plicate (Fig. 4d,e). We found a total absence of functional channels in 70% of the recordings (Fig. 4f). In the remaining 30%, only multi-channel clusters were detected (more than 20 per patch and 44 channels per patch in average).

**Figure 5:**
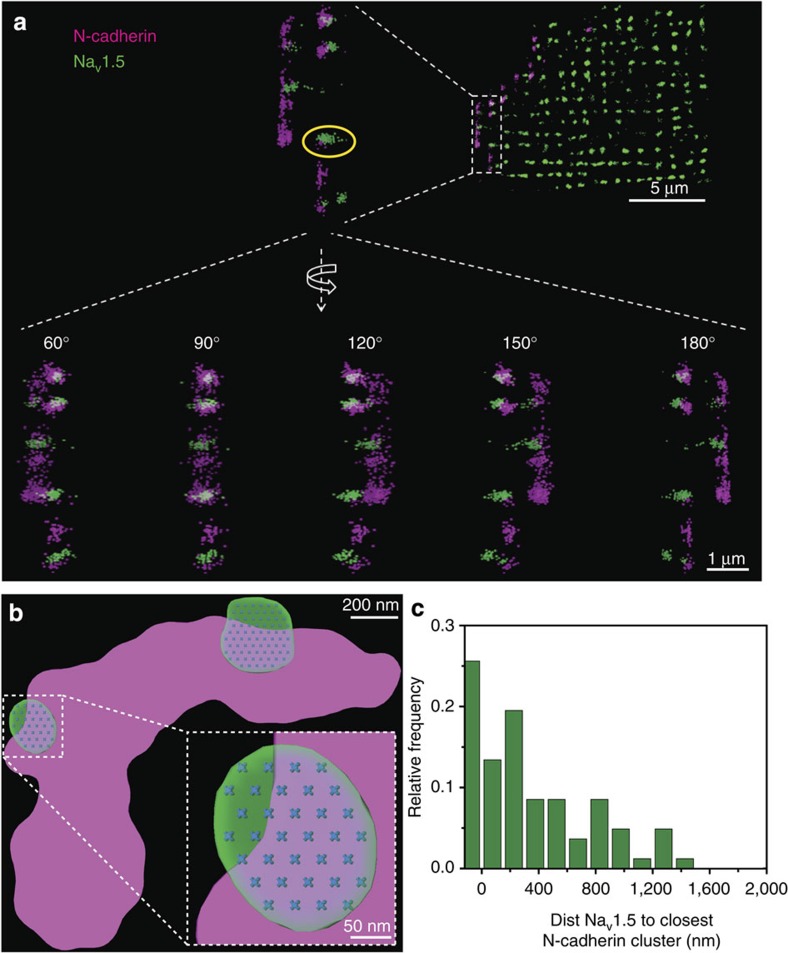
3D-SMLM images from an isolated ventricular myocyte. N-cadherin in purple, Na_V_1.5 in green (see also Methods and Supplementary Figs 7 and 8). (**a**) Region in white dotted box enlarged and shown at various angles of rotation. (top) Scale bar, 5 μm; (bottom) scale bar, 1 μm. (**b**) Enlarged image of a cluster of N-cadherin in contact with two clusters of Na_V_1.5, with the distribution of single Na_V_1.5 molecules, modelled as described in Supplementary Fig. 9). Scale bar, 200 nm. (inset) Scale bar, 50 nm. (**c**) Histogram of events. Distance between Na_V_1.5 and closest N-cadherin clusters. *N*=82.

To identify the structural correlate of the functional studies in Fig. 4, we implemented 3D-SMLM^33^ (see Supplementary Figs 7 and 8 and Supplementary Movies 1 and 2 for details). Structural features of the Na_V_1.5 clusters are presented in Supplementary Table 3 and in Fig. 5. There was a widespread distribution of cluster sizes, consequent in part to the presence of small ‘satellite' aggregates distributed in the periphery of ‘core' clusters of larger size (see cluster in yellow circle in Fig. 5a). This particular spatial arrangement was not seen in the CLEM data set, and it may have resulted from cluster de-stabilization consequent to cell dissociation. Dual-colour 3D-SMLM allowed us to detect the position of Na_V_1.5 in relation to N-cadherin (Fig. 5a and Supplementary Movies 1 and 2; structural details of N-cadherin clusters in Supplementary Table 4). Our studies found that 35% of core clusters co-localized or edged within 100 nm from N-cadherin (Fig. 5c). Most importantly, the average size of the Na_V_1.5 core clusters measured experimentally closely correlated with the predicted dimension measured by angle view patch clamp, assuming single-molecule dimensions and molecular densities similar to those reported for other systems^34^,^35^,^36^,^37^ (Fig. 5b and Supplementary Fig. 9). This distinctive convergence of functional and structural data supports the notion that the clusters detected by SMLM are the anatomical units providing functional sodium current at the ID. To examine the relation between these nanoscale observations and the microscale (that is, the overall ID structure/function), we solved the complete 3D structure of the cardiac tissue ID and compared its dimensions with those of the Na_V_1.5 clusters observed by SMLM.

### 3D ultrastructure of the ID of adult murine ventricle

**Figure 6:**
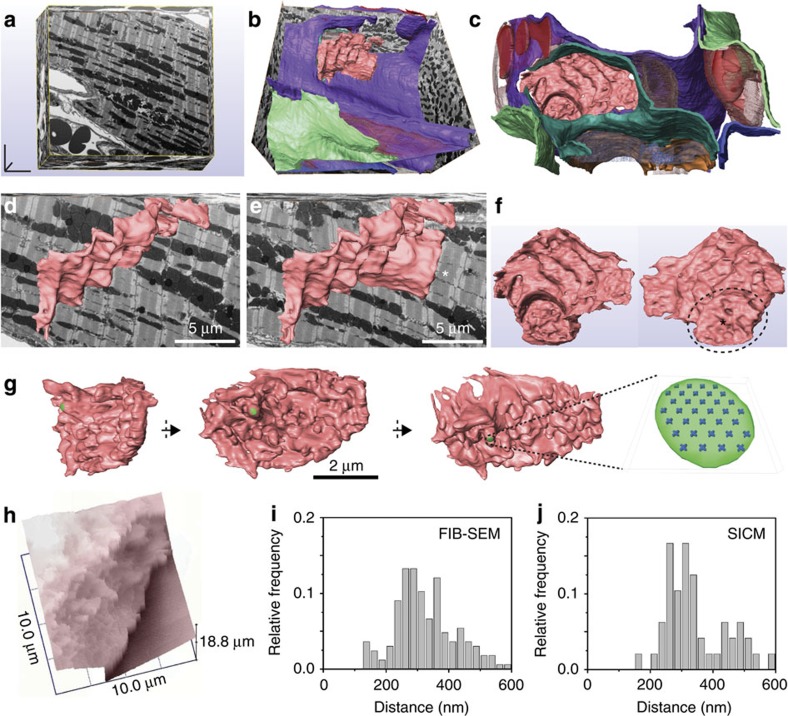
3D ultrastructure of the ID and comparative Na_V_1.5 cluster dimensions. (**a**–**g**) FIB-SEM and segmentation of a complete ID from adult ventricular tissue. (**a**) Three-dimensional cube of tissue (∼30 × 28 × 12 μm^3^; see Methods). (**b**,**c**) 3D-rendered models of structures visible after image segmentation: a complete ID (light pink), blood vessels and red blood cells (solid and transparent red), a portion of a second ID (transparent blue) and lateral membranes of up to six cells (different tones of green, white, blue, orange and violet) are visible. (**d**,**e**): 3D-rendered model of complete ID overlaid on two virtual tissue sections at different depths (2.5 μm apart from each other in the z-axis). Scale bar, 5 μm. (**f**) Complete three-dimensional view of the ID at two orientations (180° rotation). Plicate region marked with asterisk shown in more detail in **g** from three different angles. Scale bar, 2 μm. A Na_V_1.5 cluster (green), and the modelled Na_V_1.5 molecules within it (blue) are depicted in the same scale. An enlarged view of the Na_V_1.5 cluster is shown on the right. (**h**) SICM image of the cell end of an isolated ventricular myocyte. Histograms in **i**,**j** compare the peak-to-peak next neighbour distances measured in the plicate region of the FIB-SEM-resolved image and those measured by SICM, demonstrating similar periodicity in the surface foldings. *N*=166 and 48 for **i**,**j**, respectively.

We used FIB-SEM to obtain a 3D view of the ID of ventricular murine tissue. The complete 3D reconstruction (Fig. 6a–f and Supplementary Movie 3) shows the ID as a structure of steps and rises (plicate and interplicate regions). Topological analysis of the plicate region revealed a surface of regularly spaced minima and maxima (Fig. 6g) that amplified the total surface area by a factor of ∼6.5 × over that of a compressed 2D projection. Altogether, the ID occupied an estimated 10% of the total capacitive surface area of the cell, while it is estimated to carry ∼50% of total whole-cell sodium current^9^,^13^. Of note, analysis of the plicate region of the isolated cell using angle view for scanning ion conductance microscopy (SICM)^38^ revealed a topology similar to that observed in tissue (Fig. 6h–j and Supplementary Figs 10 and 11), thus suggesting adequate structural preservation of this domain after dissociation. In Fig. 6g, the Na_V_1.5 cluster displayed in Fig. 5b is overlaid on the landscape of the plicate region, and shown to occupy an area similar to that of the peak of a microplicae. Estimating an inter-cluster distance of ∼1 μm and a surface area amplification factor of 6.5 × , a 2 μm-diameter patch pipette (as used inref. 13) would include ∼19 clusters under the patch, which collectively would generate an average peak *I*_Na_ amplitude of 501 pA (44 channels per cluster, 10 pS unitary conductance and driving force of 60 mV). This number is within the range estimated directly by cell-attached macropatch (442 pA; ref. 13) and supports the idea that the functional and structural clusters reported here are responsible for the macroscopic *I*_Na_ independently recorded at the ID.

### Na_V_1.5 expression and intercellular adhesion strength

**Figure 7:**
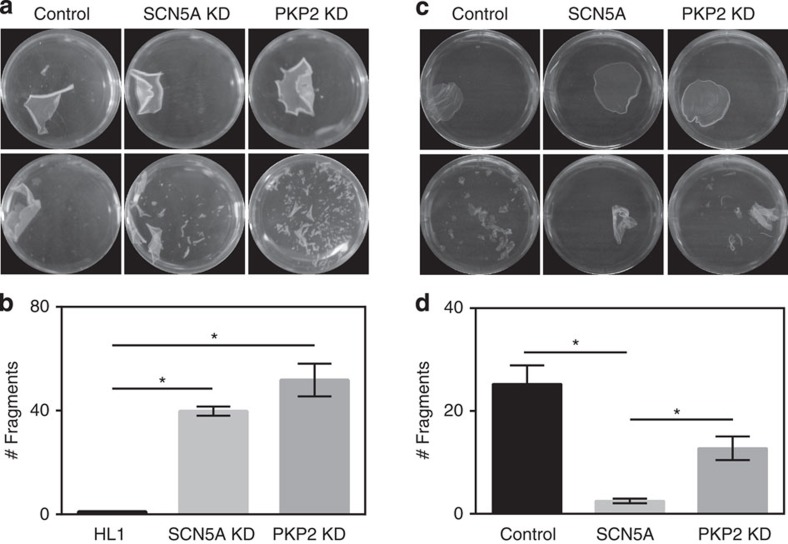
Dispase assay in HL1 cells. (**a**) Dispase treatment caused separation of the monolayer in all cells treated (upper row). Mechanical stress following dispase (bottom row) led to the breakdown of cells lacking either Na_V_1.5 (middle) or plakophilin-2 (PKP2; used as a positive control for a molecule known to be fundamental in cell–cell adhesion), but not of control cells expressing endogenous levels of both proteins (left column; see also Supplementary Fig. 12). Cell plates were placed in the same shaker, motion stopped once we saw the breakdown of the PKP2-shRNA monolayer and fragments counted for all three conditions. Plate diameter is 35 mm. (**b**) Bar graph showing the average number of fragments recorded under the three conditions tested. *N*=5 for all cases. *P<0.01 by Mann–Whitney test.

The physical proximity between two central components of adhesion and excitability (N-cadherin and Na_V_1.5, respectively) led us to hypothesize that just as adhesion molecules can affect sodium current^3^,^4^,^6^,^19^,^25^, expression of Na_V_1.5 may affect IAS. To examine this hypothesis, we implemented a dispase assay, extensively used by this and other laboratories to evaluate IAS^3^,^39^. Our results show that loss of expression of endogenous Na_V_1.5 in HL1 cells resulted in a decrease in IAS (Fig. 7). Indeed, the extent of fragmentation caused by mechanical stress following dispase treatment was much larger in cells lacking Na_V_1.5 expression than in control HL1 cells treated with a non-silencing construct (PKP2-KD cells used as a positive control; see refs 18, 19). Conversely, IAS was improved in HEK cells by exogenous expression of Na_V_1.5. (Supplementary Fig. 12).

## Discussion

We have implemented an array of nanoscale visualization techniques, combined with mathematical simulations, to demonstrate the presence of adhesion/excitability nodes at the ID region of adult ventricular tissue. Our data show that Na_V_1.5 at the intercellular membrane is organized in clusters that preferentially localize to the vicinity of N-cadherin. Angle view scanning patch clamp studies have provided a functional correlate to the anatomical observations, showing that functional sodium channels are found in distinct clusters separated by areas void of activity. The structural and functional nanoscale data, when placed in the context of the overall landscape of the ID (here solved by FIB-SEM), have indicated that the observed Na_V_1.5 clusters sufficiently explain the magnitude of the sodium current previously recorded from the ID region of adult ventricular cells^13^. Furthermore, experiments in cell culture preparations have shown that Na_V_1.5 expression impacts intercellular adhesion strength. Our results disrupt the notion of the VGSC and the adhesion complexes as independent entities. Instead, our results support a shift in paradigm whereby adhesion/excitability nodes host a protein interacting network that regulates both, excitability and adhesion between cells.

Technical considerations forced us to limit our anatomical observations to two molecules. N-cadherin is well established as a key component of the area composita and Na_V_1.5 as the sodium channel pore-forming protein, central to the function of the VGSC. Those two molecules have been independently characterized as components of multi-molecular complexes and as such, serve as marking posts not only for themselves but also for their respective associated complexes. Which molecules within those complexes interact directly and which others associate indirectly, remain to be determined. In fact, it is important to emphasize that our studies do not imply a non-covalent binding between Na_V_1.5 and N-cadherin, nor is the latter necessary for our studies to be relevant. Indeed, indirect interactions can be highly relevant from the functional standpoint. A case in point is the targeted delivery of Cx43 to the ID, which requires N-cadherin for microtubule anchoring and yet, it does not involve direct association between the two molecules^40^,^41^.

It is important to note that our CLEM studies focused only on those Na_V_1.5 clusters at the intercellular membrane. As such, we were able to analyse the relation to N-cadherin for that specific Na_V_1.5 subpopulation. This particular distinction was only possible by combining EM with SMLM. Similarly, detection of sodium channels specifically at the cell end required the development of new methods of recording (angle view scanning patch clamp). As a result of these technical efforts, we have obtained functional and anatomical data with a spatial accuracy unprecedented for these particular cardiac complexes. The implementation of these methods was justified on the basis of the specific questions being addressed (where, in the ID landscape, does Na_V_1.5 reside? How is it organized? Is the SMLM-observed Na_V_1.5 the possible source of the ID sodium current?). It should be noted that for the patch clamp data it was necessary to work in isolated cells and as such, we lost the integrity of the ID proper. The changes in sodium channel function that result from the loss of cell–cell adhesion are unknown, but likely involve a decrease in the number of functional channels at the cell membrane (see ref. 13).

Our studies showed that the relation between N-cadherin and Na_V_1.5 was deterministic. Yet, we also found Na_V_1.5 clusters at the intercellular membrane that were not positioned next to N-cadherin. This finding has important implications of its own. Indeed, standard methods of analysis (fluorescence microscopy and immunoprecipitation) cannot distinguish the association between molecules in a cluster per cluster basis and as such, do not have the sensitivity to indicate which proportion of the molecules of interest are indeed forming that association. It is sometimes assumed that if one molecule associates with another, this association extends to the entire regional pool. But the improved spatial resolution utilized in the present study strongly leads us to suggest that this is not the case. Instead, our studies indicate that Na_V_1.5 clusters, though all present in the plicate region, can be in association with more than one macromolecular complex. This may provide for a high level of versatility of sodium channel regulation, the implications of which remain unexplored.

In our CLEM experiments, we did not observe Na_V_1.5 clusters in the interplicate regions (where gap junction plaques are primarily localized) nor did we detect a preference for Na_V_1.5 clusters to occupy a particular position within the plicate domain (other than in proximity to N-Cadherin). Yet, we did observe the relation between Na_V_1.5 and N-cadherin as a deterministic event. Based on the ultrastructural information available for the dimensions of the intercellular space in the plicate region, on one hand^12^, and the mathematical modelling results estimating the necessary cleft distances for electric field-mediated propagation, on the other^15^,^16^,^17^, we would estimate that the Na_V_1.5 clusters associated with N-cadherin reported in this study would be too far away from each other to support electric field-mediated cell-to-cell propagation. Yet, the possibility cannot be completely discarded, given the limitations of our own study. Because our observations were made in thin tissue slices, it is possible that we missed analysing a particular plane in *z*, at which a subpopulation of channels may have a predilection for a particular area of the plicate domain (for example, the perinexal space^15^). Whether such a plane exists, and whether it would contain enough channels to generate the necessary electrical field for cell-to-cell propagation, is unknown.

As mentioned above, the proximity between Na_V_1.5 and N-cadherin does not necessarily imply direct binding between both molecules. In fact, we do not observe N-cadherin corralling Na_V_1.5 (in a pattern similar to what has been observed for other molecular complexes, for example, ref. 42). Previous studies have shown that the microtubule plus-end tracking protein ‘end-binding 1' (EB-1) binds to N-cadherin^40^ and to desmoplakin^43^, both components of the area composita. Other studies have also shown that Na_V_1.5 is delivered to the membrane via the microtubule network^44^. Moreover, studies in neurons have shown that, once at the membrane, the mobility of Na_V_1.5 is restricted by its scaffolding proteins, thus forming clusters at defined locations^45^,^46^. We therefore propose that the apposition of N-cadherin and Na_V_1.5 clusters is consequent to the combination of targeted delivery to the area composita, and restricted diffusion of the delivered Na_V_1.5, likely by Ankyrin-G^45^,^46^.

The presence of adhesion and excitability molecular complexes within a common hub is reminiscent in principle of the molecular organization of the node of Ranvier, where Ankyrin-G/Spectrin cores (also present in the ID) organize Na_V_1.5 clustering within a confined space shared by cell adhesion complexes^24^,^34^,^47^,^48^. The association of Na_V_1.5 and N-cadherin may be important for both, excitability and cell adhesion. Recent studies show that Na_V_1.5 function is sensitive to local mechanical forces^49^,^50^. As such, its proximity to mechanical junctions can provide it with mechanical stability and limited membrane deformation during the contractile cycle. On the other hand, our results suggest that Na_V_1.5 can contribute to intercellular adhesion strength. As such, the existence of both Na_V_1.5 and N-cadherin clusters within the same adhesion/excitability node may explain why mutations in Na_V_1.5 can lead not only to arrhythmias but also to cardiomyopathies that, in some cases, can severely compromise the cardiac function^29^. Our data open the possibility of Na_V_1.5 as a novel modulator of cell adhesion and mechanical integrity in the heart.

## Methods

### Tissue sample preparation and SMLM imaging for CLEM

Mice were anaesthetized with sodium pentobarbital, perfused with 4% paraformaldehyde in phosphate-buffered saline (PBS) and then killed by excision of the heart. The perfused heart was cut into 1-mm^3^ sections and placed in a solution containing 2% paraformaldehyde in PBS and 0.1% glutaraldehyde for 4 h at 4 °C. After washing with PBS, the tissue was embedded with 10% gelatin, infused with sucrose and cryosectioned at 80 nm on formvar-carbon-coated EM finder grids (Electron Microscopy Sciences, Hatfield, PA). The grids were then processed for immunostaining as follows: samples were washed with double-distilled water (ddH_2_O) before being incubated in PBS containing 0.12% glycine for 5 min. Blocking was done with PBS containing 2% bovine serum albumin and 0.2% gelatin for another 5 min. Primary antibodies rabbit Na_V_1.5 (Sigma) (1:50) and monoclonal mouse anti-N-cadherin (BD Bioscience) (1:100) were diluted in blocking solution and incubated overnight at 4 °C. Primary antibodies were then washed with PBS and secondary antibodies were incubated for 1 h at room temperature. Secondary antibodies used were: Mouse Alexa Fluor 647 (Life Technologies) (1:5,000) and Rabbit Alexa Fluor 568 (Life Technologies) (1:5,000). Before mounting the sample for imaging, fiducial markers were added to the sample by placing the grid on a drop of a solution containing gold particles (Nanopartz Inc) (1:200). Grids were mounted between a slide and a glass coverslip with imaging buffer (200 mmol l^−1^ mercaptoethylamine and an oxygen scavenging system: 0.1 mg ml^−1^ glucose oxidase, 0.02 mg ml^−1^ catalase and 0.8% (wt/wt) glucose). Samples were imaged in a custom-built microscopy set up^30^ equipped with an HCX PL APO × 63 NA=1.47 OIL CORR TIRF objective followed by achromatic × 2 tube lens magnification, yielding a total magnification of × 126. Total internal reflection fluorescence or highly inclined illumination modes with solid/state lasers 532 and 640 nm were used to excite the samples and improve the signal-to-noise ratio. Two-colour movies containing a minimum of 2,000 frames were processed using an ImageJ macro routine based on the QuickPALM plugin. For this analysis, the two-colour image was split into its two separate colour channels, each reconstructed at 20 nm per pixel using the following QuickPALM parameters full-width half-maximum=4 and signal/noise=2.00. The reconstructed super-resolved images of each channel were then superimposed to generate a two-colour super-resolved image. The mapping error in the super-resolved image was 20 nm. Cluster analysis was performed using ImageJ software. Images were processed with a smoothing filter, adjusted for brightness and contrast and filtered to a threshold to obtain a binary image. Cluster detection and parameters were obtained using the function ‘Analyze particles.'

### Sample processing and EM imaging for CLEM

After SMLM imaging, grids were recovered from the imaging chamber and washed thoroughly with PBS to remove the mounting medium. Grids were then post fixed with 1% glutaraldehyde for 5 min, washed with PBS and distilled water. Samples were then contrasted and embedded in a mixture of 3% uranyl acetate and 2% methylcellulose in a ratio of 1:9. For EM imaging, grids were examined and the same regions imaged by SMLM were localized. Low and high magnification (from × 1,250 to × 19,500) TEMs were acquired using a Philips CM-12 electron microscope (FEI; Eindhoven, The Netherlands) equipped with a Gatan (4 k × 2.7 k) digital camera (Gatan, Inc., Pleasanton, CA).

### Correlation of fluorescence and TEM images

A coarse assessment of the region of interest was done using the square marks in the finder grids and other references in the tissue such as foldings or edges. Then, the Control Point Selection Tool in Matlab (Mathworks) was used to select the position of the added fiducial markers visible in the EM and in the fluorescent images. Their coordinates allowed us to calculate the transformations needed to bring both images in register^51^. An affine transformation was typically applied, to account for possible distortion effects in the tissue during processing and imaging for TEM.

### Analysis of CLEM images

Using the EM image as reference, a line was drawn following the membrane at the ID and a mask was created allowing 500 nm on either side of the ID line in order to select only the clusters localized in this region for further analysis. Cluster detection was performed^30^. Briefly, in order to standardize measurements and reduce the possibility of errors, cluster parameters (centroid coordinates, edges, area and circularity among others) as well as distance between clusters (Na_V_1.5 to Na_V_1.5, N-cadherin to N-Cadherin and Na_V_1.5 to N-cadherin clusters) were obtained using the ImageJ function ‘Analyze particles' and a script written in Python that utilized the image processing packages ‘scikit-image' and ‘mahotas.' The Origin program was used for data management. Extensive validation of the SMLM methods is presented in the online supplement of a previous publication^30^.

### Monte Carlo simulations of cluster placement for CLEM data

The experimentally observed two-dimensional (2D) clusters of Na_V_1.5 and N-cadherin were modelled as ellipses. The size distribution of these ellipses corresponded to that of the experimental data. These clusters were placed in a 2D matrix (a box) that approximated the area immediately surrounding the ID region (500 nm on either side of the ID line, as per the experimentally determined region of interest). There were 11 correlative images from the experimental data set. The combined ID line lengths and cluster counts from each of these images were used to simulate 11 corresponding boxes on which to draw the ellipses. Simulations placing the clusters in this group of 11 boxes were run 1,000 times, and the corresponding distances between clusters were measured and averaged over all simulations. Two scenarios were modelled; first a random placement of both Na_V_1.5 and N-cadherin ellipses in the boxes. Second, an ‘attraction factor' was introduced, in which the placement of each Na_V_1.5 ellipse was made more likely to occur overlapping an N-cadherin ellipse. In both models, the N-cadherin ellipses were first drawn in the box, by randomly selecting a major/minor axis length from the list of experimentally observed N-cadherin clusters, and then randomly selecting a position and rotation angle for the corresponding ellipse from a uniform distribution. If overlap occurred between N-cadherin ellipses, a new random position was chosen until all ellipses could be placed. In the random model, the Na_V_1.5 ellipses were placed in a similar manner, by selecting randomly from a uniform distribution. In the ‘attraction' model, the Na_V_1.5 ellipses were placed using a non-uniform probability distribution. A parameter *f* (ranging from 2 to 8) determined the ratio between the probability for placement at a position overlapping an N-cadherin ellipse and the probability for placement with no overlap (*f*=1 corresponds to the random case described above). The procedure was as follows: first a Na_V_1.5 ellipse and position was chosen randomly. If this ellipse did not overlap with an N-cadherin ellipse, a random number r was chosen (0–1). If *r*>1/*f*, the old position was discarded and a new random position was chosen, continuing in this manner until all ellipses were placed. All coding for the simulations was done in Python, and the Python libraries pandas and matplotlib were used for the data manipulation and graphing of results. The simulations were performed at the High Performance Computing Facility of the Center for Health Informatics and Bioinformatics at New York University Langone Medical Center. Computer code can be made available upon request.

### Particle averaging

Based on the coordinates of the cluster centroids detected in the SMLM images analysis, individual clusters of overlapping Na_V_1.5 and N-cadherin located to the ID membrane were extracted from the SMLM images in 100 × 100 pixel^2^ boxes (2 μm × 2 μm). A total of 50 boxes containing the co-localized (edge-to-edge distance ≤0) Na_V_1.5 and N-cadherin clusters were aligned and averaged. Original SMLM clusters were first binerized and splitted in their two channels. An initial rough alignment was performed using the green channel signal. Transformations obtained in this step were then applied to the red channel signal of the particles and a second step of alignment was performed. Once optimal transformations were applied, the two channels of the particles were merged back and their signals summed up to get the final averages. A scoring function based on cross-correlation that adopts its maximum value when all particles are in register was used to obtain optimal shifts and rotations. All the processing was performed using MATLAB and ImageJ.

### Random walk simulations

A random walk model was used to simulate the movement of an example regulatory molecule in a 3D rectangular cuboid representing a region surrounding a cluster of Na_V_1.5 channels localized to the intercellular membrane. An immobilized ligand (‘Na_V_1.5 proteins') was placed at the surface of a cuboid, and the regulatory molecules (the ligates) were permitted to randomly move in discrete steps within the confines of the space. The length and width of the modelled surface was determined based on the average centroid-to-centroid distance between Na_V_1.5 clusters from the experimentally measured 2D SMLM data: 1,066 nm. The distance represented by each step in the model was considered to be 33 nm, a magnitude corresponding to the estimated pore-to-pore distance between Na_V_1.5 channels (see refs 24, 34 andSupplementary Fig. 9). Therefore, the number of steps in the *x* and *y* direction was calculated as


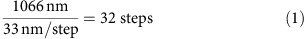


The height of the space was set to 16 to make it large enough so that edge effects would not affect the results. The total volume represented by the rectangular cuboid was then measured as


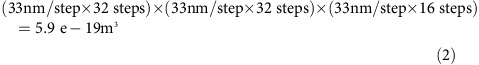


To take a particular example, parameters for the ligate–ligand interaction were those previously measured for the interaction between Calmodulin (CaM) and the C-terminal domain of Na_V_1.5 (ref. 52). Based upon a concentration of 6 μM for CaM, the number of molecules placed in the cuboid with the above volume was calculated as 2,127. The number of Na_V_1.5 channels placed on the surface of the cuboid was set at 44 based on the patch clamp data from this study. The channels were positioned in both clustered arrangement and an equally distributed arrangement at the surface position (Supplementary Fig. 6). The CaM molecules were initially randomly arranged in the cuboid and then at each time step were allowed to move with equal probability to any adjacent position, with the exception of a particle immediately adjacent to a Na_V_1.5 channel, as explained below. In addition, particles were allowed to bind if occupying the same position as a Na_V_1.5 channel, and also to dissociate if currently bound to a channel. Two variables naturally occurred in the system: (1) the probability that a CaM molecule will bind to a Na_V_1.5 channel once it occupies the same position; and (2) the probability that a CaM molecule will dissociate from a Na_V_1.5 channel once bound. A third variable was also introduced, an ‘attraction factor': when a CaM molecule was one step away from an adjacent Na_V_1.5 channel, this attraction factor increased the probability that the particle would move to a position containing the channel. This variable represented the fact that modulators of Na_V_1.5 can be brought into proximity by common molecular partners. Since the actual values for these probabilities were unknown, simulations were run with an array of probabilities for the binding and dissociating variables to determine which combinations would produce *K*_d_ measurements near the average of experimentally measured value of 88 nM (apoCaM) and 132 nM (Ca2+/CaM) for the CaM Na_V_1.5 complex^52^. The random walk was performed with 500 time steps, this being adequate to produce a stable system. Once an appropriate set of probabilities were determined, for both the presence and absence of the attraction factor, the random walk simulations were run 1,000 times for each combination and the two distribution scenarios for the Na_V_1.5 channels were compared. The coding for these simulations was written in Python, utilizing the package matplotlib for the initial visualization of simulation results.

### Adult mouse myocyte dissociation for SICM

All animal procedures related to patch clamp studies conformed to the UK Animals (Scientific Procedures) Act 1986 Imperial College London Ethical Review Committee and the project license authorized these studies in accordance with the United Kingdom Home Office Animals (Scientific Procedures) Act 1986. Cardiomyocytes were isolated by the Langendorff perfusion method^53^. Briefly, adult mouse were anaesthetized with 5% isoflurane-95% O_2_ and then killed by cervical dislocation. Hearts were quickly extracted and placed in Tyrode solution using aortic cannulation with the Langendorff setting. The isolated hearts were then perfused sequentially with low calcium and an enzyme (collagenase type V, 1 mg ml^−1^; proteinase type XXIV 0.36 mg ml^−1^; and hyaluronidase 0.6 mg ml^−1^; Sigma-Aldrich) solution. Ventricles were cut into small pieces and gently minced with a Pasteur pipette. Calcium concentration was then increased gradually to normal values. After isolation, cardiomyocytes from left ventricle were plated on laminin coated coverslips and left to adhere for at least 45 min before the start of experiments. Cardiomyocytes were used on the same day of isolation. Cells were washed once with the external recording solution and mounted on the microscope stage for electrophysiological recordings.

### Angle view SICM

SICM is a non-contact scanning probe microscopy technique based on the principle that the flow of ions through the tip of a nanopipette filled with electrolytes decreases when the pipette approaches the surface of the sample^54^,^55^,^56^. The result is a 3D topography image of live cells with resolution of up to 20 nm^57^. In this study, we used a variant of SICM called hopping probe ion conductance microscopy^38^ mounted in a PatchStar micromanipulator (Scientifica, UK) that allows us to adjust the angle at which the scanning nanopipette approaches the cell. The angle can be adjusted in a range of 0–90° to the surface; an angle of 33° was experimentally selected as optimal for scanning and patch. The whole-angle hopping probe ion conductance microscopy set up can be integrated into an upright optical microscope with × 40 water immersion objective, implemented on a software platform that controls the ICnano sample scan system^56^ (Ionscope). With this set up we are able to resolve the ID area with nanoscale resolution. Borosilicate glass nanopipettes pulled from 1.0 mm outer diameter, 0.4 mm ID capillary were used in all experiments. Axopatch 200B patch clamp amplifier (Axon Instruments; Molecular Devices) was used to measure the pipette current as well as to record ion channel activity. Cell-attached currents were digitized using Digidata 1440A and a pClamp 10 data acquisition system (Axon Instruments; Molecular Devices).

### Cell-attached electrophysiological recordings

After resolving the region of the cardiomyocyte previously forming the ID, and positioning the pipette in the area of interest, the non-contact mode of SICM was turned off and the pipette was lowered as in a conventional patch clamp. After a slight suction, a gigaseal was obtained and electrophysiological recordings performed using the cell-attached patch clamp configuration. Recordings were performed at room temperature using the following solutions; external solution containing in (mmol l^−1^): 145 KCl; 1 MgCl_2_; 1 CaCl_2_; 2 EGTA; 10 Glucose; 10 HEPES; and pH 7.4 with KOH; internal recording solution containing in (mmol l^−1^): 135 NaCl; 0.4 NaH_2_PO_4_; 1 MgCl_2_; 5.4 KCl; 1CaCl_2_; 5.5 glucose; 5 HEPES; 20 TEA-Cl; 0.2 CdCl_2_; 10 CsCl; 10, 4-AP; and pH 7.4 with NaOH. The pipette used for cell-attached recordings had an average resistance of 33±2 MΩ. Currents were recorded using an Axopatch 200B amplifier, controlled and monitored using pClamp 10. To generate a current–voltage (*I*–*V*) relationship, the membrane under the patch was held at a voltage of −120 mV and incremental steps of 10 mV were applied from −80 to 0 mV. Data were low-pass filtered at 1 kHz using the built-in Bessel filter of the amplifier and sampled at 20 kHz.

### Experimental set up for 3D SMLM data acquisition

SMLM imaging was done using a custom-built fluorescence microscope (Leica DMI3000 and Olympus IX51) configured for total internal reflection fluorescence and highly inclined excitation modes as described before^30^. To achieve three-dimensional imaging, a cylindrical lens (focal length=10,000 nm, Melles Griot) was placed in the imaging path^33^. To maintain focus over time and stabilize *z*-drift in the microscope focus during acquisition, a CRISP Autofocus System (Applied Scientific Instrumentation) was mounted on the system.

### Calibration of *z*-localization for 3D SMLM imaging

Fluorescent beads (0.1 μm diameter, Life Technologies) were adsorbed on a coverglass surface at a low density. A *z*-stack image was generated by acquiring images at 10 nm *z*-intervals, ranging from −500 to 500 nm considering 0 when the beads were in focus. A 3D calibration table with the ‘PSF Width minus Height' values over the *z*-position was created using the plugin QuickPALM on ImageJ^58^ (Supplementary Fig. 7).

### Adult myocyte dissociation for 3D SMLM experiments

All procedures were in accordance with New York University guidelines for animal use and care (IACUC Protocol 130812-01 to MD approved on 08/13/2014) and conformed to the Guide for the Care and Use of Laboratory Animals published by the US National Institutes of Health (NIH Publication 58-23, revised 1996). Experiments were carried out in C57BI/6 adult mice of both genders (3–6 months of age). Adult mouse ventricular myocytes were obtained by enzymatic dissociation following standard procedures. Briefly, mice were injected with 0.1 ml heparin (500 IU ml^−1^ intraperitoneally) 20 min before heart excision and anaesthetized by carbon dioxide inhalation. Deep anaesthesia was confirmed by lack of response to otherwise painful stimuli. Hearts were quickly removed from the chest and placed in a Langendorff column. The isolated hearts were perfused sequentially with low calcium, and collagenase solution (Worthington). Atria were discarded and ventricles were gently minced with a Pasteur pipette. Calcium concentration was reintroduced gradually to normal values. After isolation, cardiomyocytes were plated on laminin coated coverslips or dishes and left to adhere for at least 30 min before the start of experiments.

### Adult myocyte 3D SMLM imaging

Cells plated in 18-mm circular coverslips were fixed in 4% paraformaldehyde and permeabilized with 0.1% Triton in PBS. Blocking was done in PBS containing 2% glycine, 2% bovine serum albumin, 0.2% gelatin, and 50 mM NH_4_Cl for 30 min. Primary antibodies rabbit Na_V_1.5 (Sigma) (1:50) and monoclonal mouse anti-N-cadherin (BD Bioscience) (1:100) were diluted in blocking solution and incubated overnight at 4 °C. Primary antibodies were then washed with PBS and secondary antibodies were incubated for 1 h at room temperature. Secondary antibodies used were: Mouse Alexa Fluor 647 (Life Technologies) (1:5,000) and Rabbit Alexa Fluor 568 (Life Technologies) (1:20,000). SMLM imaging for 3D reconstructions was performed as described at the beginning of this Methods section (tissue sample preparation and imaging in SMLM for CLEM)^30^. Movies containing 2,000 frames were acquired and used to reconstruct 3D super-resolved images using QuickPALM^59^.

### Analysis of the 3D SMLM data

Cluster analysis was performed using ImageJ software. *Z*-stack images were processed with a smoothing filter and adjusted for brightness and contrast^30^. For Na_V_1.5 cluster analysis, only the clusters localized at the cell end (within 500 nm of the N-cadherin line) were considered for analysis. Cluster detection and parameters were obtained using the ImageJ plugin ‘3D Objects Counter.'^60^ Same Python script used before was adapted to allow 3D analysis.

### FIB-SEM sample preparation

The protocol was modified from Wilke *et al.*^61^ Mice were anaesthetized by carbon dioxide inhalation and perfused with 0.1 M phosphate buffer containing 2% paraformaldehyde and 2.5% glutaraldehyde. The hearts were then excised and post fixed in the same fixative solution at 4 °C overnight. After washing with 0.1 M phosphate-buffered saline for 30 min at room temperature, the tissue was placed in phosphate buffer containing 2% OsO_4_/1.5% potassium ferrocyanide for 1 h at room temperature, washed three times for 5 min in double-distilled H_2_O (ddH_2_O) at room temperature and then placed in a filtered solution of 1% thiocarbohydrazide (EMS) in ddH_2_O for 20 min at room temperature to allow for additional osmium-staining. The tissue was then washed three times in ddH_2_O and then placed in 2% aqueous OsO_4_ for 30 min at room temperature. Finally, the tissue was washed three times in ddH_2_O and placed in 1% aqueous uranyl acetate at 4 °C overnight. The next day, tissue was washed three times in ddH_2_O. *En bloc* lead staining was performed to enhance membrane contrast^62^. A lead aspartate solution was made by dissolving 0.066 g of lead nitrate in 10 ml of 0.003 m aspartic acid. The pH was adjusted to 5.5 with 1 N KOH, and the solution was placed in a 60 °C oven for 30 min. The lead aspartate solution was filtered, and the tissue was stained at 60 °C for 30 min. It was determined that this enhanced osmium-staining protocol, combined with *en bloc* lead staining, was critical for enhancing membrane contrast. The sample was then washed three times in ddH_2_O, dehydrated in a series of ethanol solutions (30, 50, 70, 85, 95 and 100%; 10 min each, on ice) and then placed in ice-cold dry acetone for 10 min, followed by 10 min in acetone at room temperature. The sample was then gradually equilibrated with Durcupan ACM Araldite embedding resin (Electron Microscopy Sciences, EMS, PA) by placing in 25% Durcupan/acetone for 2 h, 50% Durcupan/acetone 2 h, 75% Durcupan/acetone for 2 h, and 100% Durcupan overnight. The tissue was then embedded in fresh 100% Durcupan and placed in a 60 °C oven for 48 h to allow Durcupan polymerization and complete the embedding procedure.

### FIB-SEM data acquisition

The sample was trimmed and thin sections were cut on slot grids to identify the area of interest. The sample block was then mounted on the s.e.m. sample holder using double-sided carbon tape (EMS). Colloidal silver paint (EMS) was used to electrically ground the exposed edges of the tissue block. The entire surface of the specimen was then sputter coated with a thin layer of gold/palladium and the tissue imaged using back scattered electron (BSE) mode in a FEI Helios Nanolab650 dual beam s.e.m. Images were recorded after each round of ion beam milling using the s.e.m. beam at 2 keV and 100 pA with a working distance of 2.5 mm. Data acquisition occurred in an automated way using the Auto Slice and View G3 software. The raw images were 4,096 × 3,536 px^2^, with 20 nm slices viewed at a 60° angle. Each raw image had an horizontal field width (HFW) of 32 μm, therefore the raw data had the following dimensions: *X*: 7.8125, nm per pixel; *Y*: 9.02 nm per pixel and *Z*: 20 nm per slice. A stack of 606 slices was aligned and assembled for further analysis.

### FIB-SEM data analysis

A volume of roughly 30 × 28 × 12 μm^3^ dimensions was obtained from the tissue block. Upon inspection of the virtual slices, a complete ID (roughly 13 × 11 × 7 μm^3^ in size), blood vessels, red blood cells, a portion of a second ID and lateral membranes of up to six cells present in the area were segmented and their 3D-rendered models obtained. This way a realistic 3D model of the region was generated, where the spatial interrelations of all the different structures of interest could be seen in high detail. Surface area of the entire ID as well as of its most upward facing plicate region (with dimensions roughly 3 × 3 × 5 μm^3^) was determined. The plicate region was revealed to form a structure of peaks and valleys. Certain degree of regularity in their distribution was observed, so, to further explore this fact, 3D distances between the peaks and their closest neighbour at both sides of the ID as well as their 2D distances upon projection of their coordinates into a single 2D plane parallel to the interface between the two apposing cells were calculated. A binary image where the positions of the 2D projected peaks were marked as white Gaussian circles over a black background was created and its 2D fast-Fourier transform obtained. Plots with the intensity profile of several representative regions of this image were generated. Segmentation and visualization was performed in Amira (Visage Imaging, San Diego, CA). ImageJ^58^ and Matlab (Mathworks, Natick, MS, USA) equipped with the Image Processing toolbox were used for quantitative analysis.

### Dispase assay

Two different cell lines were used: HL1 cells, a cardiac muscle cell line derived from AT-1 mouse atrial cardiomyocyte tumour linage and previously utilized in our laboratory^18^,^19^,^63^ and HEK cells, derived from human embryonic kidney cells (ATCC). To generate the Na_V_1.5-deficient HL1 cell line (Na_V_1.5-KD), a Lenti-*SCN5A* mouse shRNA clone (TRCN0000069005) was packaged using a Trans-Lentiviral Packaging Kit and Calcium Phosphate (GE Dharmacon). The HL1 cells were infected with the packaged lentivirus. A separate line was generated that expressed a non-silencing Lenti vector; this cell line was used as a control. Cells were selected with Claycomb media containing puromycin. The method was similar to that used in our previous publications^18^,^19^. A stable PKP2-deficient HL1 cell line (PKP2-KD)^19^ was used as a positive control for weak intercellular strength. Separate experiments were carried out in HEK cells stably expressing Na_V_1.5, kindly provided by Dr Silvia Priori, characterized in^64^ and used to determine IAS as in^65^. The assay was performed as previously described^3^,^39^. Briefly, cells were plated at confluency in six-well plates. After 24 h, cells were incubated with 2.4–4.8 U ml^−1^ Dispase at 37 °C until cell monolayers detached from the bottom of the dish. Mechanical stress to a comparative set (for example, HL1 control versus Na_V_1.5 KD versus PKP2KD) was induced by placing the plates on an orbital shaker. Motion was interrupted once we observed disruption of the PKP2KD monolayer (used as a positive control) and the number of fragments in all three groups, counted. For the HEK293 cell experiments, motion was interrupted once we observed fragmentation of the control line.

## Additional information

**How to cite this article:** Leo-Macias, A. *et al.* Nanoscale visualization of functional adhesion/excitability nodes at the intercalated disc. *Nat. Commun.* 7:10342 doi: 10.1038/ncomms10342 (2016).

## Supplementary Material

Supplementary Information Supplementary Figures 1-12 and Supplementary Tables 1-4

Supplementary Movie 1 3D-SMLM of an adult isolated myocyte labeled for Na_V_1.5 (green) and N-Cadherin (purple). The movie displays a Z-stack of images and then a rotation of the cell around the Y axis to allow visualization of the cell end in three dimensions.

Supplementary Movie 2 Close-up view and rotation of a group of Na_V_1.5 and N-Cadherin clusters (green and purple, respectively) at the cell end of the myocyte shown in Supplementary Movie 1.

Supplementary Movie 3 FIB-SEM of a block of ventricular tissue containing a complete intercalated disc. Aligned stack of images and 3D rendered models of the ID and other surrounding structures obtained by segmentation.

Supplementary Movie 4 Plicate region of the ID. Same region as that contained within the dashed circle traced in Supplementary Movie 3. The three-dimensional model was obtained by segmentation.

Supplementary Movie 5 Rotation of the 3D rendered model corresponding to the ID plicate region shown in Supplementary Movie 4.
