# Erratum: Nanoscale visualization of functional adhesion/excitability nodes at the intercalated disc

**DOI:** 10.1038/ncomms10919

**Published:** 2016-02-22

**Authors:** Alejandra Leo-Macias, Esperanza Agullo-Pascual, Jose L Sanchez-Alonso, Sarah Keegan, Xianming Lin, Tatiana Arcos, Feng-Xia-Liang, Yuri E Korchev, Julia Gorelik, David Fenyö, Eli Rothenberg, Mario Delmar

Nature Communications 6: Article number: 10342 10.1038/ncomms10342 (2015); Published: 01202016; Updated: 02222016

The author Eli Rothenberg was incorrectly omitted from the list of corresponding authors in this Article and authors Julia Gorelik and David Fenyö were incorrectly listed as corresponding authors. Furthermore, Julia Gorelik, David Fenyö, Eli Rothenberg and Mario Delmar should have been listed as having jointly supervised the work. The correct information for correspondence is: ‘Correspondence and requests for materials should be addressed to E.R. Eli.Rothenberg@nyumc.org) or to M.D. mario.delmar@nyumc.org).'

