# Supplementary material for: Nanoscale visualization of functional adhesion/excitability nodes at the intercalated disc
Source: Nat Commun. 2016 Jan 20;7:10342. doi: 10.1038/ncomms10342 (PMC4735805; doi:10.1038/ncomms10342)
Supplement: Supplementary Information — Supplementary Figures 1-12 and Supplementary Tables 1-4 [file ncomms10342-s1.pdf]

SUPPLEMENTARY INFORMATION

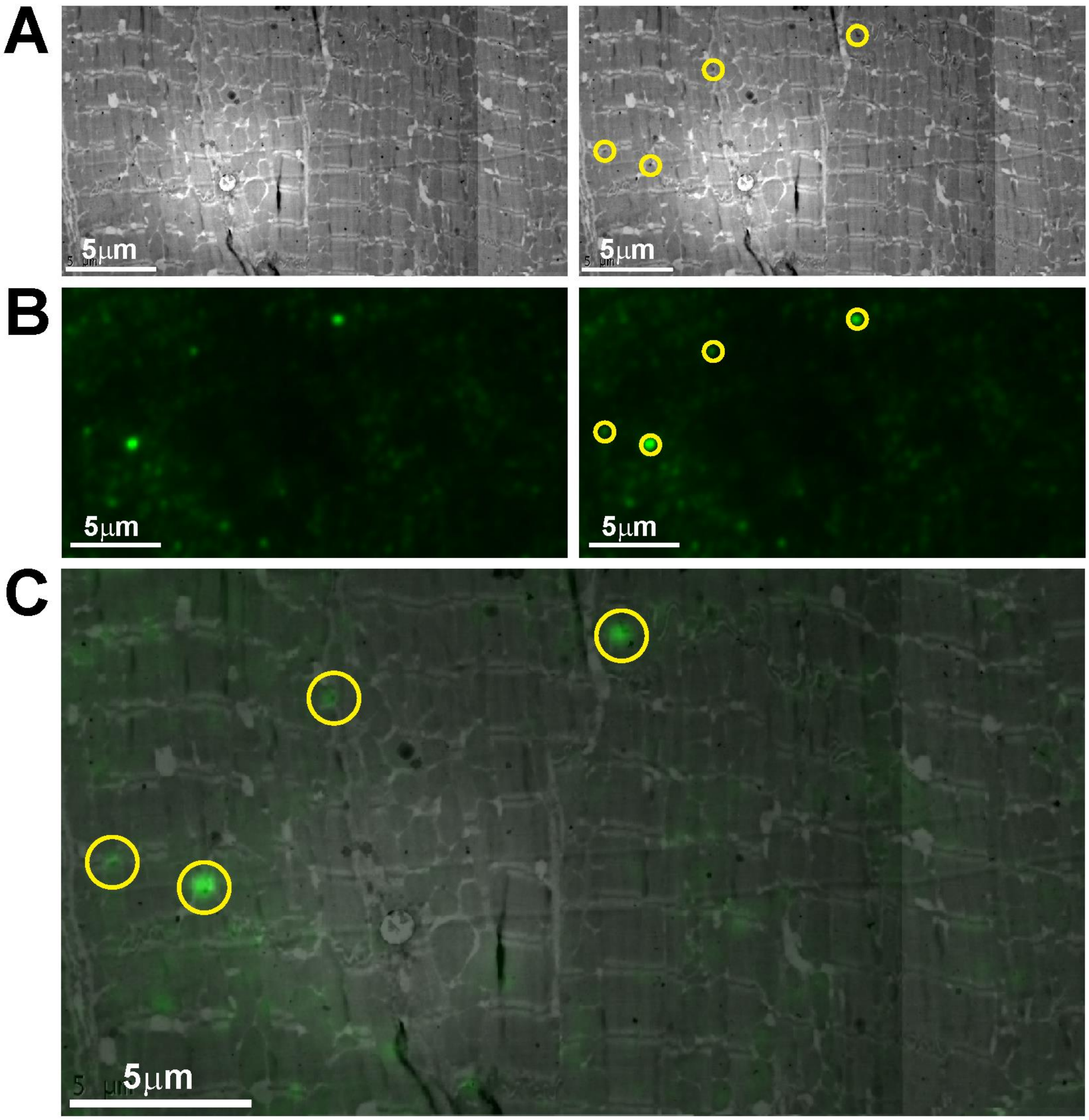

Supplementary Figure 1: Method for alignment of EM and SMLM images for CLEM using gold beads as fiducial markers. Thin slices (~80 nm) of adult ventricular mouse tissue were mounted on Finder grids. Gold beads (200 nm) were also deposited on the preparation, and their position in the EM image detected as electron dense, circular structures (A). The beads also fluoresced in the green spectrum and as such, were detectable under fluorescence microscopy (B). Beads that were detected under both configurations (EM and fluorescence microscopy) served as unbiased fiduciary markers for alignment of both images (C).

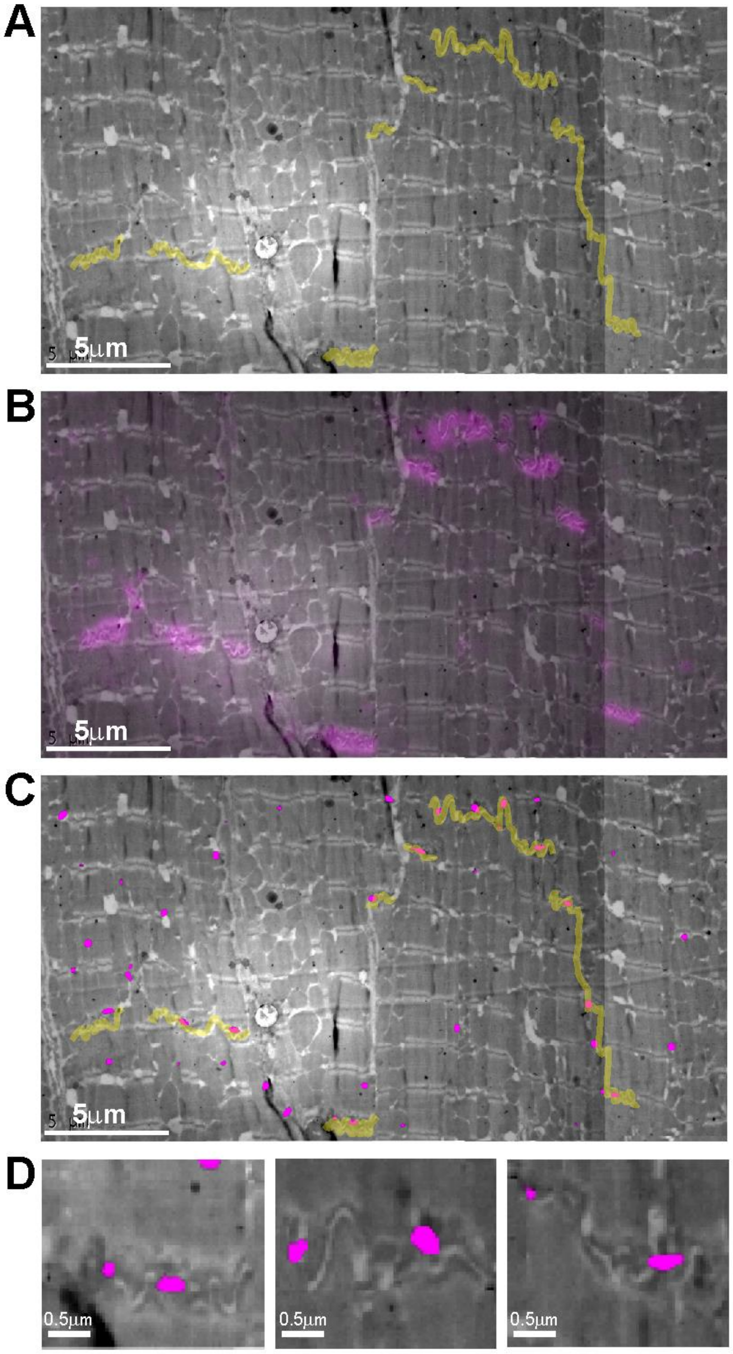

Supplementary Figure 2: Detection of the N-Cadherin signal on the aligned CLEM images. The preparation described in Supplementary Figure.1 was treated to detect the N-Cadherin immunoreactive signal on the EM landscape. In panel A) we have traced the intercellular membrane to aid visualization. The N-Cadherin fluorescent signal after alignment is presented in B). Panel C) shows an overlay of the N-Cadherin signal detected by SMLM (red) on the EM landscape, with the intercellular membranes traced in yellow. Three areas are enlarged in D) to show the overlay of the SMLM -detected N-Cadherin signal on the intercellular membrane identified by EM.

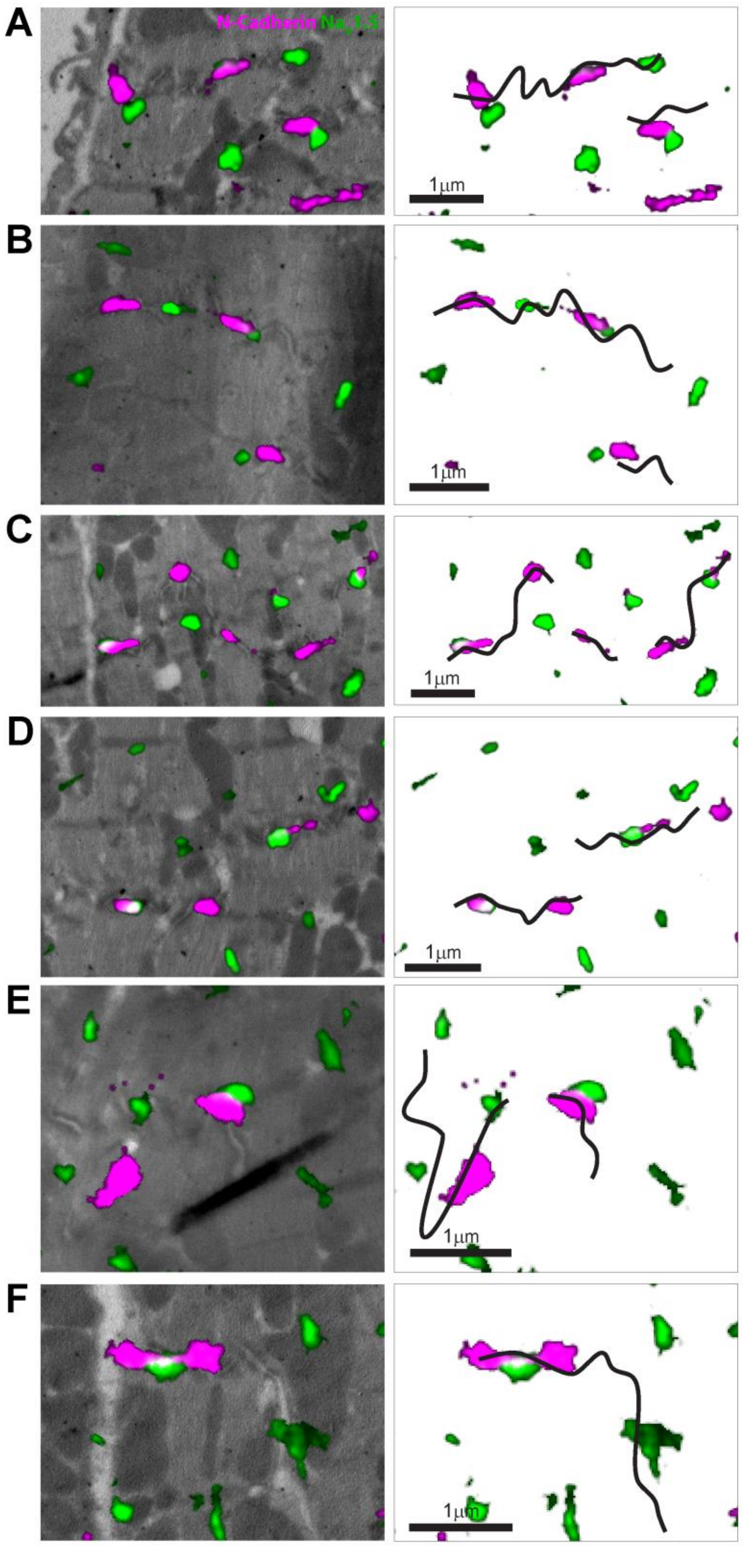

Supplementary Figure 3: Examples of CLEM of Na_V1.5 and N-Cadherin in adult ventricular tissue. Left panels show a series of CLEM images obtained as per the method described in Supplementary Figure.1-2. For the right panels, only the traced intercellular membrane (black lines), and the N-Cadherin and Na_V1.5 signals are displayed (purple and green, respectively). Each panel shows an independent example.

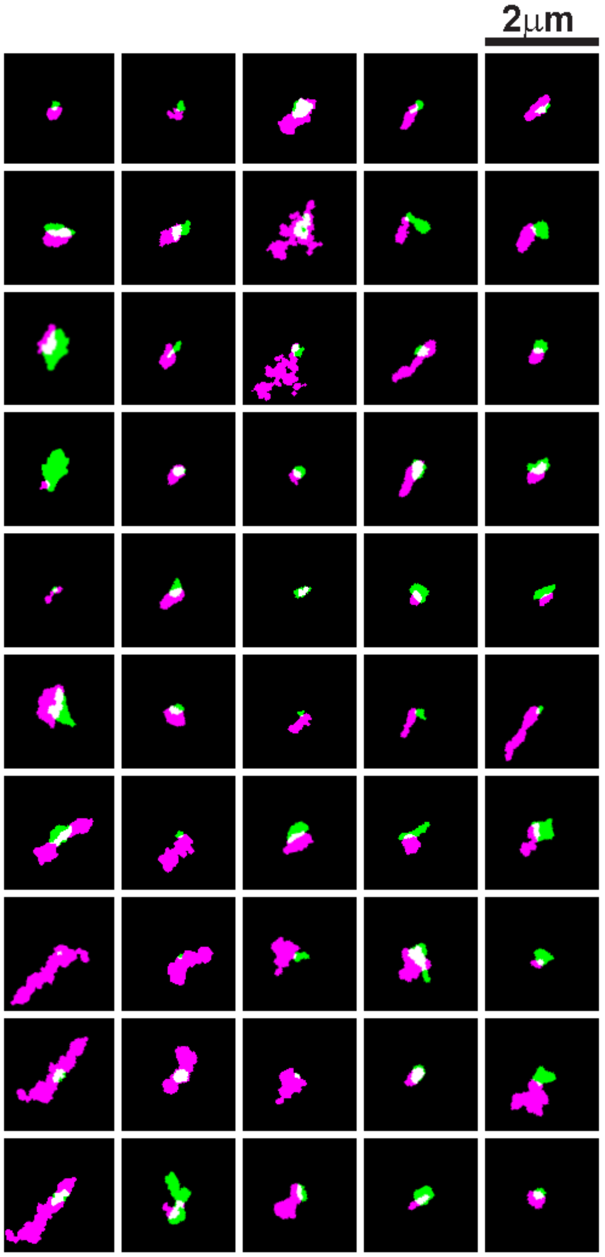

Supplementary Figure 4: Particle averaging analysis. Only clusters where $Na_V1.5$ and N-Cadherin co-localized (even if partially) and were located on the intercellular membrane, were included (a total of 50 separate boxes (2μm x 2 μm); 49 separate Nav1.5 particles; one particle co-localized with two different N-cadherin clusters). Particle alignment and averaging was carried out as described in the Methods.

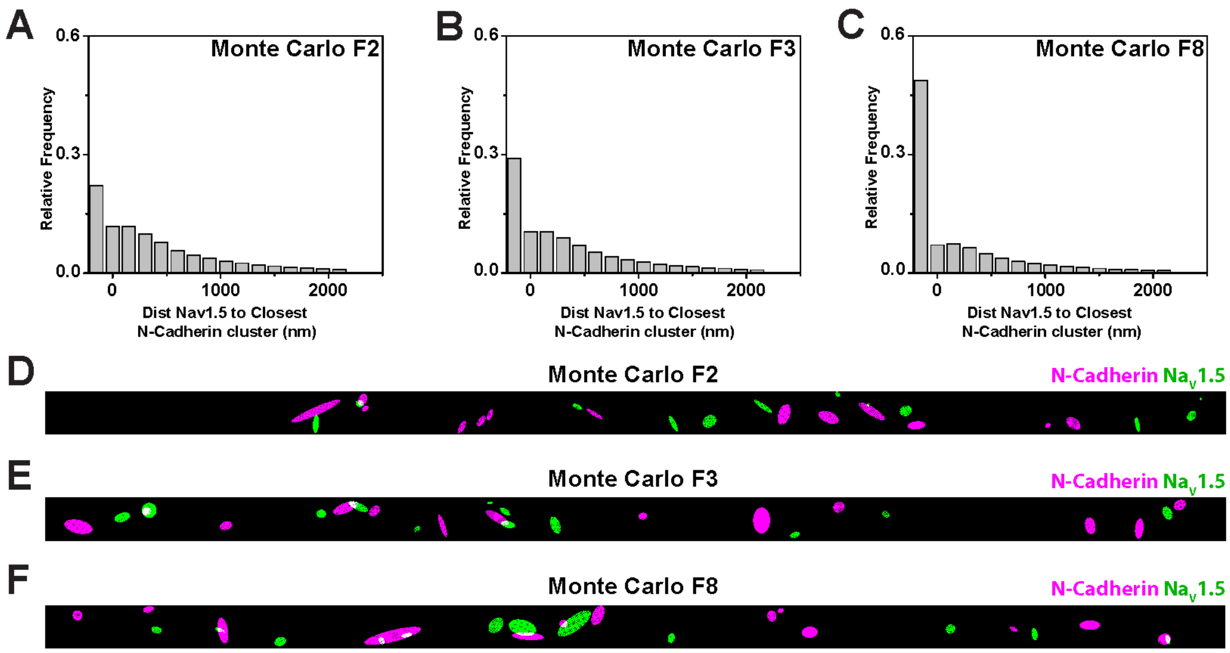

Supplementary Figure 5: Monte Carlo simulations. A-F) Monte Carlo simulations to determine the likelihood of Na_V1.5- N-Cadherin co-localization in the intercellular membrane (Fig. 1 of main manuscript and Supplementary Figure.1-4) using a random versus deterministic model. A-C) Histograms depicting the number of instances (relative to total; n=147) over 1000 simulations at which the Na_V1.5 cluster is detected at a given distance from the closest N-Cadherin. F2, F3 and F8 refer to the attraction factor employed for the simulation. Examples of the simulated clusters in all three conditions are presented in D-F).

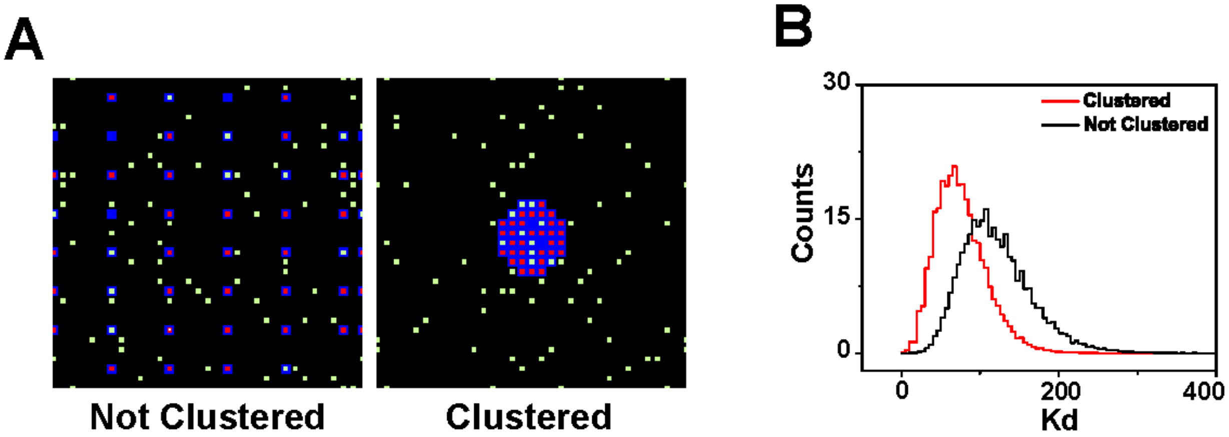

Supplementary Figure 6: Random walk simulations. A) Blue/green squares represent immobilized ligands (e.g., $Na_V1.5$) presented with a soluble ligate (e.g., a binding partner such as calmodulin). Blue alone is the $Na_V1.5$ unit by itself. Red indicates that ligand and ligate are interacting. Two configurations were studied: one where the ligands were distributed homogeneously over the entire surface (left; not clustered) and one where the ligands were concentrated within a cluster (right). B) The effect of clustering was evaluated by assessing shifts in the K_d of the reaction. Each model consisted of 500 steps, and resulted in a stable K_d value for the system. A total of 1000 models were run in order to produce a probability distribution (i.e., each histogram includes 1000 events). Data show that the interaction between $Na_V1.5$ and a regulatory molecule is significantly facilitated by the clustering of $Na_V1.5$. Additional details in the Methods.

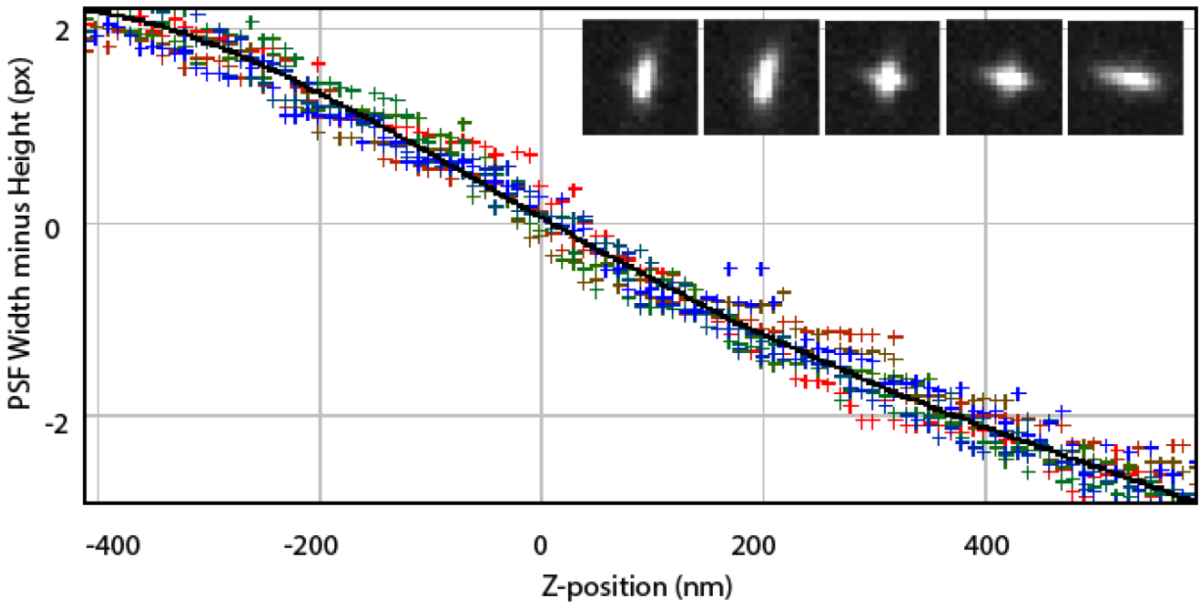

Supplementary Figure 7: Calibration curve for the astigmatism imaging method used to generate the 3D-SMLM images of $Na_V1.5$ and N-Cadherin presented in Figure 3 of the manuscript. Width minus height of the Point Spread Function (PSF) relates to the z position of an emitter (100 nm diameter beads). Examples of astigmatic images used are shown in the inset.

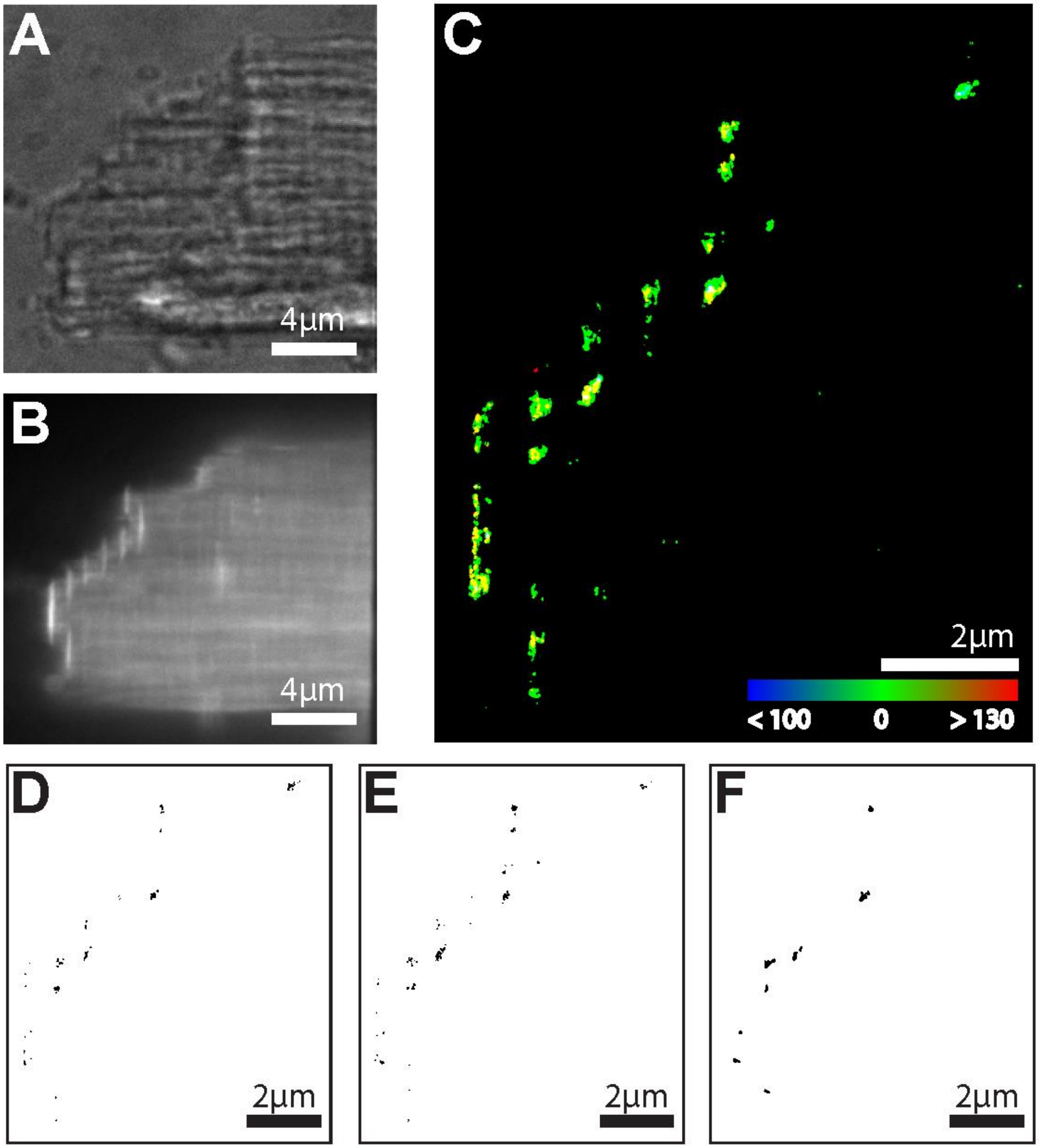

Supplementary Figure 8: N-Cadherin imaging by 3D-SMLM at the cell end of an adult ventricular myocyte. A) Phase contrast image; B) TIRF image of the N-Cadherin signal. C) 3D-SMLM data collected from the same sample as in A-B). Depth is color-coded as per the bar in the lower right margin. D-F) Three different image planes revealing the ability to resolve signals in the z axis.

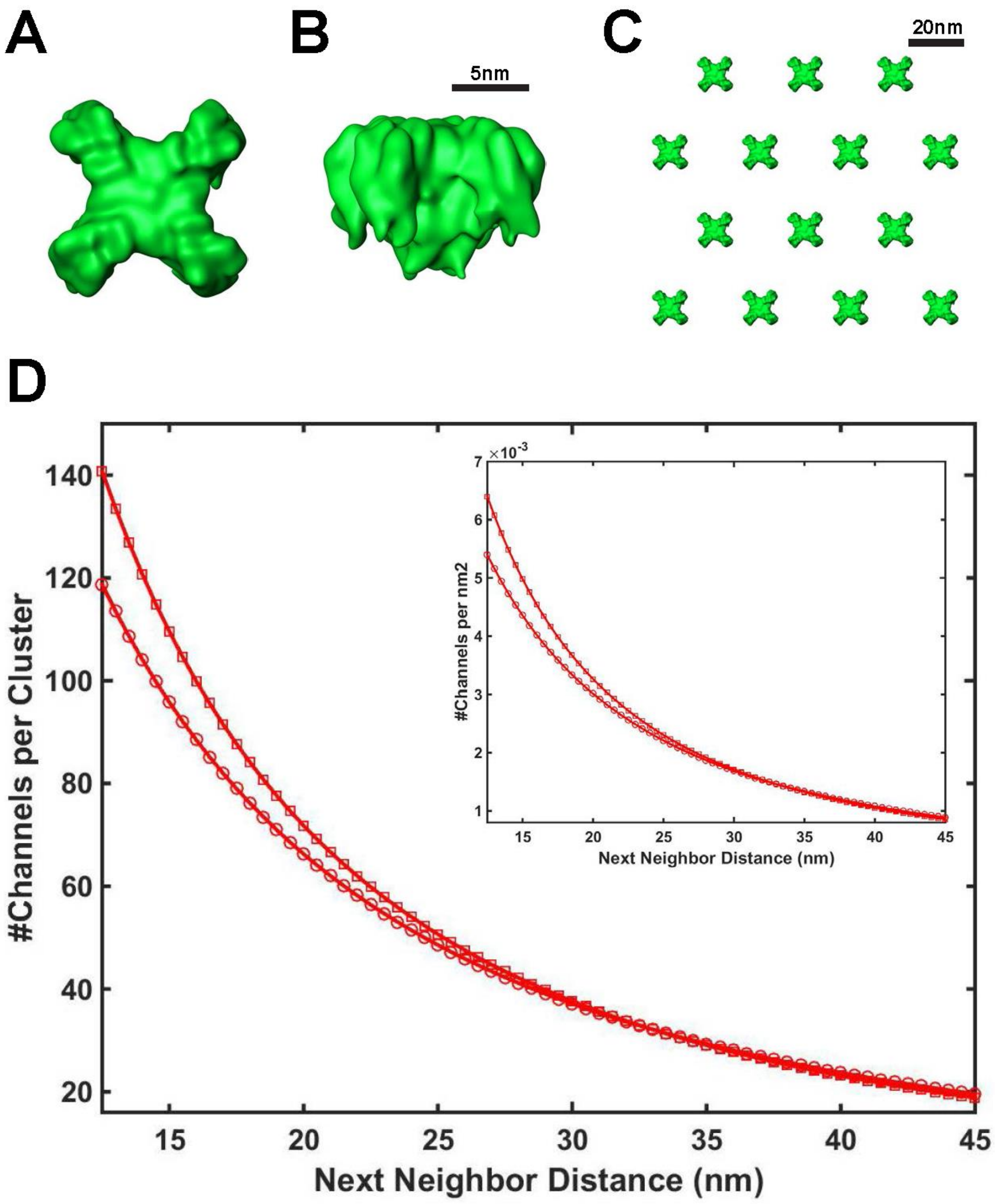

Supplementary Figure 9: Molecular modeling used to estimate the number of $Na_V1.5$ molecules that fit within the core $Na_V1.5$ clusters resolved by 3D-SMLM (see Figure 3 of the main manuscript). A and B show the physical dimensions of a sodium channel, based on the structure of the bacterial NavAb oligomer and the particle size (sodium channel unit) detected at the node of Ranvier. An hexagonal array of NavAb units is shown in C). The plots in D) represent the number of channels per cluster (or per unit of area) as a function of next neighbor distance. Studies in node of Ranvier indicate that next neighbor distance in that system is between 25 and 40 nm. Using those numbers, we would estimate that 25 to 50 channels would fit within a $Na_V1.5$ cluster of average size. This number is consistent with the number of channels per patch detected by angle view scanning patch clamp and reported in Figure 4 of the manuscript.

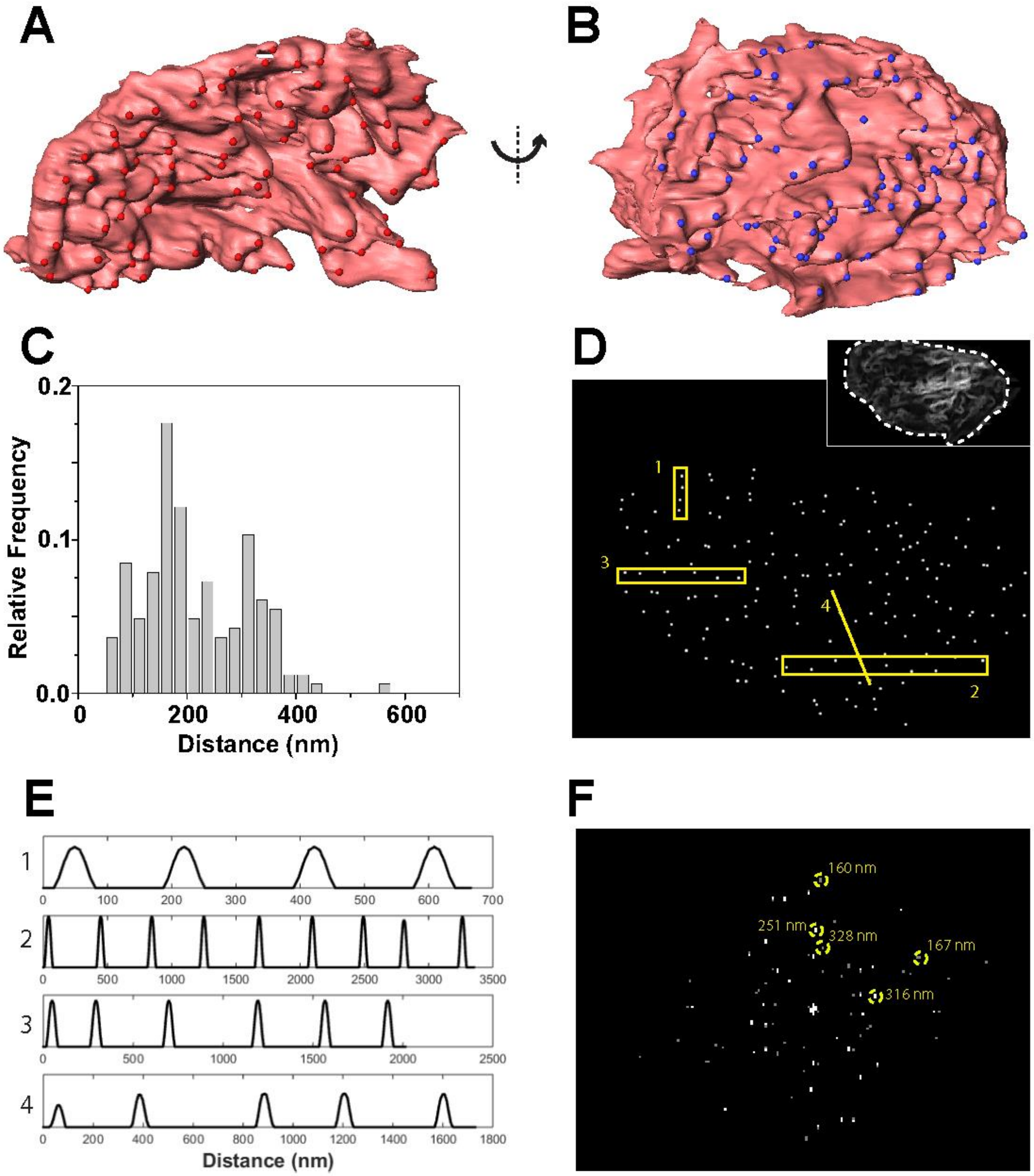

Supplementary Figure 10: Ultrastructure of the plicate region of the intercalated disc by FIB-SEM. Segmentation analysis from the sample depicted in Figure 6 of the manuscript. A and B) Red and blue dots localize the peaks and valleys of the segmented structure. Image in B) is a 180-degree rotation of the one in A). C): Histogram of the distance from each point (either red or blue) and closest neighbor when all peaks and valleys are projected into a single 2D plane parallel to the interface between the two apposing cells. N=166. D) Binary image where the positions of the 2D projected peaks were marked as white Gaussian circles over a black background. Inset inside dashed line shows the area occupied by the plicate region mask when projected into the plane of the interface between the cells. E) Intensity profile plots of regions marked in D. F) Fast Fourier Transform of image in D).

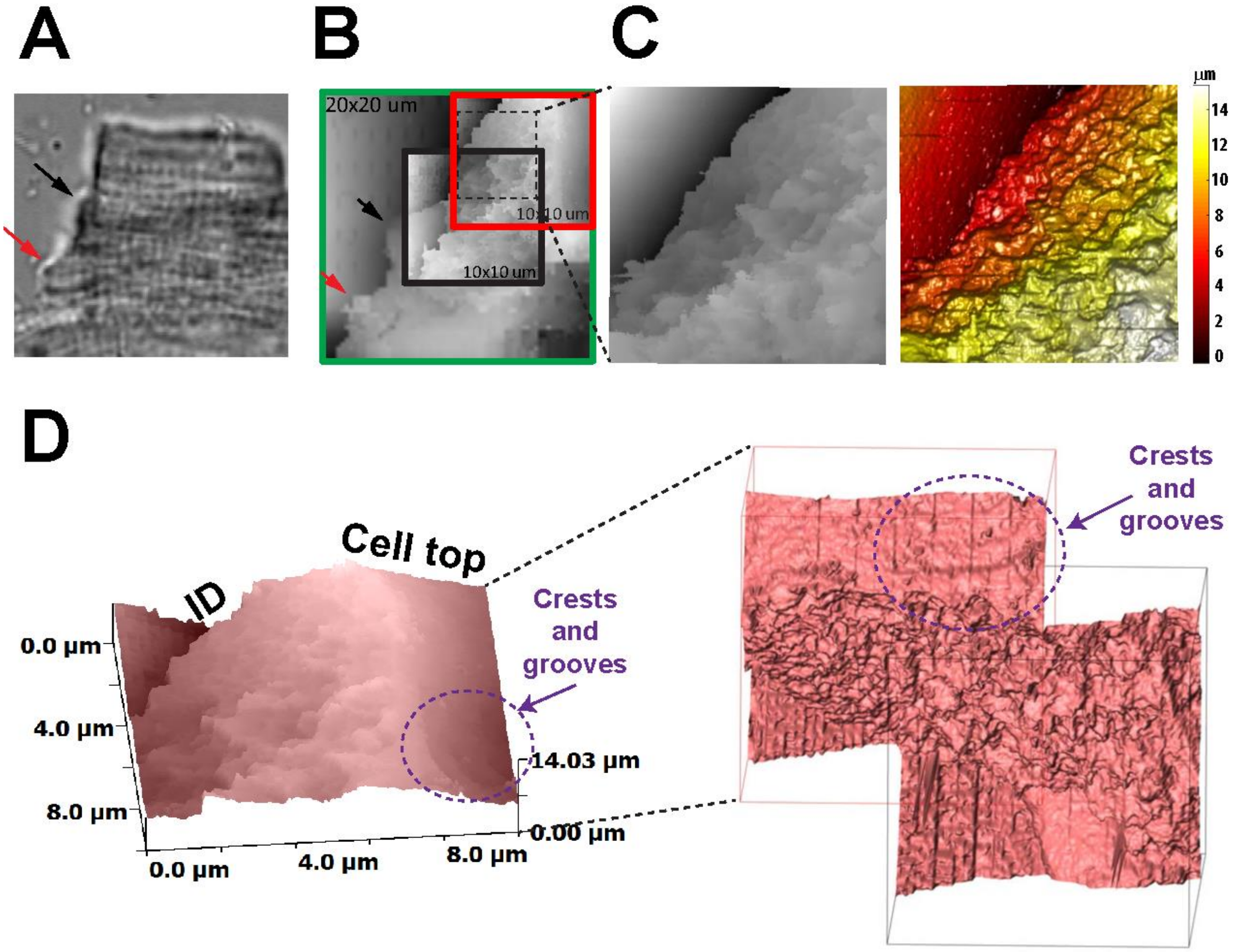

Supplementary Figure 11: Angle-view SICM of the myocyte cell end. A) Phase contrast image, examined in B) and C) by the SICM probe. Black and red arrows in A) and B) serve to orient the SICM data with the phase contrast image. A scan at high resolution (~20 nm) yielded the images in the frames in B), one of which is enlarged in C), with a depth profile (calibration bar on the right). The top of the cell is better visualized in D) (region inside red square in B), where crests and grooves can be distinguished. The rough surface of the cell end, organized in peaks and valleys, can be appreciated on the images of the right side of the panel D, which correspond to regions inside the red and black square in B).

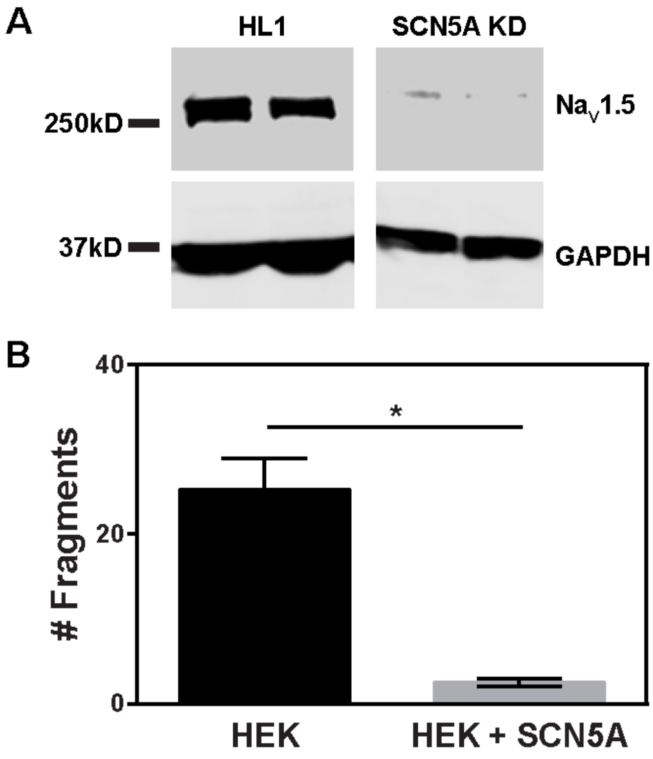

Supplementary Figure 12: Dispase assay in HEK cells. A) Confirmation of Na$_V$1.5 silencing in HL1 cells by Western blot. GAPDH was used as loading control. B) HEK293 monolayers and HEK293 monolayers stably expressing *SCN5A* were subjected to mechanical stress after treatment with dispase. Expression of Na$_V$1.5 significantly improved intercellular adhesion strength. Graph bar show number of fragments after mechanical stress. n = 20-16, *p < 0.01 by Mann-Whitney test.

Supplementary Table 1: Summary of properties of **Na_V1.5 clusters** detected in **CLEM** images of cardiac tissue.

All clusters (11 separate images):

	N	Mean	SE	Minimum	Median	Maximum
**Area**	147	35575	2455	2800	28000	174400
**Circularity**	147	0.75	0.01	0.31	0.75	1
**Edge Distance Closest Na_V1.5**	118	814	72	45	636	3747
**Centroid Distance Closest Na_V1.5**	118	1030	72	131	946	4055

*8 clusters of Na_V1.5 without a neighboring N-Cadherin were included to measure area/circularity

No overlap with N-Cadherin:

	N	Mean	SE	Minimum	Median	Maximum
**Area**	90	30088	2599	2800	24400	102000
**Circularity**	90	0.76	0.02	0.31	0.76	1
**Edge Distance Closest Na_V1.5**	77	765	85	60	599	3489
**Centroid Distance Closest Na_V1.5**	77	971	85	131	865	3612

Overlap with N-Cadherin:

	N	Mean	SE	Minimum	Median	Maximum
**Area**	49	44294	5204	2800	36000	174400
**Circularity**	49	0.73	0.03	0.35	0.73	1
**Edge Distance Closest Na_V1.5**	41	907	132	45	653	3748
**Centroid Distance Closest Na_V1.5**	41	1139	133	173	969	4055

*8 clusters of Na_V1.5 without a neighboring Na_V1.5 were included to measure area/circularity

Supplementary Table 2: Summary of properties of **N-Cadherin** clusters detected in **CLEM** images of cardiac tissue.

All clusters:

	N	Mean	SE	Minimum	Median	Maximum
**Area**	158	65129	4085	10000	53000	319200
**Circularity**	158	0.64	0.02	0.11	0.67	1
**Edge Distance Closest N-Cadherin**	122	610	47	40	429	2617
**Centroid Distance Closest N-Cadherin**	122	957	48	273	828	2897

*12 clusters of N-Cadherin without a neighboring Na_V1.5 were included to measure area/circularity

No overlap with Na_V1.5:

	N	Mean	SE	Minimum	Median	Maximum
**Area**	92	54243	3759	10000	48400	183200
**Circularity**	92	0.66	0.02	0.23	0.70	1
**Edge Dist Closest N-Cadherin**	85	619	58	40	418	2617
**Centroid Dist Closest N-Cadherin**	85	945	58	273	781	2897

Overlap with Na_V1.5:

	N	Mean	SE	Minimum	Median	Maximum
**Area**	44	95391	10811	11600	72800	319200
**Circularity**	44	0.55	0.03	0.10	0.52	0.91
**Edge Dist Closest N-Cadherin**	37	589	83	40	444	1994
**Centroid Dist Closest N-Cadherin**	37	987	86	314	899	2362

*7 clusters of N-Cadherin without a neighboring N-Cadherin were included to measure area/circularity

Supplementary Table 3: Summary of properties of **Na_V1.5** clusters detected in **3D-SMLM** images of isolated adult myocytes.

Volume range 150.000 – 1.000.000nm^3:

	N	Mean	SE	Minimum	Median	Maximum
**Volume (nm^3)**	588	307320	7336	152000	232000	984000
**Surface (nm^2)**	588	47045	1017	21600	38400	150400
**Nb of obj. voxels**	588	38	1	19	29	123
**Nb of surf. voxels**	588	38	1	18	29	115
**Mean dist. to surf. (nm)**	588	47	1	27	44	108
**Edge Distance Closest Na_V1.5**	588	205	12	0	80	1714
**Centroid Distance Closest Na_V1.5**	588	268	13	0	138	1810
**Edge Distance Closest N-Cadherin**	588	448	16	0	344	1874
**Centroid Distance Closest N-Cadherin**	588	553	16	39	452	1951

Volume range > 1.000.000nm^3:

	N	Mean	SE	Minimum	Median	Maximum
**Volume (nm^3)**	82	4.07E+06	0.50 E+06	1.02E+06	1.94E+06	2.92E+07
**Surface (nm^2)**	82	385297	34090	105600	232400	1.46E+06
**Nb of obj. voxels**	82	509	63	127	243	3655
**Nb of surf. voxels**	82	391	38	115	223	1677
**Mean dist. to surf. (nm)**	82	103	4	61	90	199
**Edge Distance Closest Na_V1.5**	82	424	54	0	135	1406
**Centroid Distance Closest Na_V1.5**	82	642	63	0	354	1810
**Edge Distance Closest N-Cadherin**	82	365	43	0	234	1430
**Centroid Distance Closest N-Cadherin**	82	608	54	0	572	1780

Supplementary Table 4: Summary of properties of **N-Cadherin** clusters detected in **3D-SMLM** images of isolated adult myocytes.

Volume range 150.000 – 1.000.000nm^3:

	N	Mean	SE	Minimum	Median	Maximum
**Volume (nm^3)**	2881	288036	3064	152000	224000	1000000
**Surface (nm^2)**	2881	45196	449	20000	36800	152800
**Nb of obj. voxels**	2881	36	0	19	28	125
**Nb of surf. voxels**	2881	36	0	18	28	124
**Mean dist. to surf. (nm)**	2881	48	0	26	44	132
**Edge Distance Closest N-Cadherin**	2881	103	2	40	63	1858
**Centroid Distance Closest N-Cadherin**	2881	177	3	41	146	1985
**Edge Distance Closest Na_V1.5**	2881	651	9	0	537	1904
**Centroid Distance Closest Na_V1.5**	2881	754	10	0	646	1999

Volume range > 1.000.000nm^3:

	N	Mean	SE	Minimum	Median	Maximum
**Volume (nm^3)**	173	7.08E+06	0.68 E+06	1.00E+06	3.03E+06	4.41E+07
**Surface (nm^2)**	173	792869	65859	134400	393600	4.46E+06
**Nb of obj. voxels**	173	884	85	125	379	5512
**Nb of surf. voxels**	173	780	69	121	369	4884
**Mean dist. to surf. (nm)**	173	136	3	65	125	276
**Edge Distance Closest N-Cadherin**	172	265	31	40	56	1653
**Centroid Distance Closest N-Cadherin**	172	559	40	90	332	1948
**Edge Distance Closest Na_V1.5**	173	483	32	0	375	1640
**Centroid Distance Closest Na_V1.5**	173	798	38	0	739	1960
